# National and subnational burden of stroke in Iran from 1990 to 2019

**DOI:** 10.1002/acn3.51547

**Published:** 2022-04-08

**Authors:** Aida Fallahzadeh, Zahra Esfahani, Ali Sheikhy, Mohammad Keykhaei, Sahar Saeedi Moghaddam, Yeganeh Sharifnejad Tehrani, Negar Rezaei, Erfan Ghasemi, Sina Azadnajafabad, Esmaeil Mohammadi, Sogol Koolaji, Sarvenaz Shahin, Nazila Rezaei, Bagher Larijani, Farshad Farzadfar

**Affiliations:** ^1^ Non‐Communicable Diseases Research Center, Endocrinology and Metabolism Population Sciences Institute Tehran University of Medical Sciences Tehran Iran; ^2^ Department of Biostatistics University of Social Welfare and Rehabilitation Sciences Tehran Iran; ^3^ Feinberg Cardiovascular Research Institute Feinberg School of Medicine, Northwestern University Chicago Illinois 60611 USA; ^4^ Endocrinology and Metabolism Research Center, Endocrinology and Metabolism Clinical Sciences Institute Tehran University of Medical Sciences Tehran Iran

## Abstract

**Background:**

Data on the burden of stroke and changing trends at national and subnational levels are necessary for policymakers to allocate recourses appropriately. This study presents estimates of the stroke burden from 1990 to 2019 using the results of the Global Burden of Disease (GBD) 2019 study.

**Methods:**

For the GBD 2019, verbal autopsy and vital registration data were used to estimate stroke mortality. Cause‐specific mortality served as the basis for estimating incidence, prevalence, and disability‐adjusted life years (DALYs). The burden attributable to stroke risk factors was calculated by a comparative risk assessment. Decomposition analysis was applied to determine the contribution of population aging, population growth, and changes in the age‐specific incidence rates.

**Results:**

In 2019, the number of prevalent cases, incident cases, and deaths due to stroke in Iran were 963,512; 102,778; and 40,912, respectively. The age‐standardized incidence rate (ASIR) and the age‐standardized death rate (ASDR) decreased from 1990 to 2019. Of national stroke ASDRs in 2019, 44.7% (35.7–54.7%) were attributable to hypertension and 28.8% (15.2–57.4) to high fasting plasma glucose. At the subnational level, the trend of the stroke incidence and mortality rate decreased in all provinces. Stroke was responsible for 4.48% of total DALYs in 2019 (3.38% due to ischemic stroke, 0.87% due to intracerebral hemorrhage, and 0.22% due to subarachnoid hemorrhage).

**Conclusion:**

ASIR and ASDR of stroke are decreasing nationally and subnationally; however, the number of incident cases and deaths are increasing in all SDI quintiles, possibly due to population growth.

## Introduction

Stroke is one of the major causes of death and disability worldwide, remaining the third leading cause of death and disability combined in 2019.^1^ The stroke‐related deaths increased from 4.6 million in 1990 to approximately 6.6 million in 2019, which is responsible for 11.59% of global mortality.^1^, ^2^ Moreover, 87% of both stroke‐related deaths and disability‐adjusted life years (DALYs) appear in low‐ and middle‐income countries.^3^, ^4^ In the Middle East and North Africa (MENA), stroke was reached from the fourth cause of death in 1990 to the second cause of death in 2019. Likewise, in Iran, stroke was reached the second cause of death in 2019 from the sixth cause of death in 1990.^5^

Iran, similar to other countries, is committed to achieve sustainable development goals (SDG), which was presented by the United Nations in 2015.^6^ According to the SDG 3.4, by 2030, it is expected that mortality due to noncommunicable diseases reduces by one‐third; indeed, large‐scale epidemiological studies are needed to examine the goal achievement. Therefore, studies at national and subnational levels are a priority in stroke research to estimate the real burden of stroke and to tackle the current knowledge gap on stroke burden in the country. One of the major limitations for making policy and tracing the progress of stroke prevention in developing countries is the lack of high‐quality data on stroke epidemiology. The results of this study were extracted from the Global Burden of Disease (GBD) study. The GBD study has provided a portrait of incidence, mortality, and DALYs of 369 diseases at the global, regional, and national levels.^1^, ^7^

Several studies have been estimated the burden of stroke in different countries. In China, stroke became the leading cause of years of life lost (YLL), and the absolute numbers and crude rate of stroke burden increased from 1990 to 2019.^8^ According to previous systematic reviews that evaluated the burden of stroke in the MENA region (consists of approximately 21 countries in southwest Asia and north Africa including Iran), there was an increase in stroke incidence and mortality rates in this region^9^; however, the incidence of stroke was shown to be lower than that in most developed western countries.^10^

National and Subnational Burden of Diseases, Injuries, and Risk Factors (NASBOD) study was conducted to estimate patterns of stroke mortality from 1990 to 2015 at national and subnational levels in Iran.^11^, ^12^ NASBOD study focused only on stroke mortality and did not provide data on the incidence, prevalence, risk factors, and burden of stroke. Despite this well‐conducted study, still there is a lack of practical evidence in order to help policymakers, in terms of risk factors and epidemiological variables, such as YLL, years lived with disability (YLD), and DALYs. This study was conducted to evaluate data on the incidence, mortality, and burden of stroke in Iran between 1990 and 2019, which is an essential step for policymaking and resource allocation.

## Methods

### Overview

This study was conducted in favor of Guidelines for Accurate and Transparent Health Estimates Reporting (GATHER), which defines best‐reporting methods for studies that calculate health estimates.^13^ We retrieved the publicly available results from the GBD 2019 study, which can be retrieved from the GBD in the “Causes” section under the GBD code “B.2.3,” including ischemic stroke “B.2.3.1,” intracerebral hemorrhage “B.2.3.2,” and subarachnoid hemorrhage “B.2.3.3” as subtypes.^5^ GBD 2019 estimated each epidemiological quantity of interest—incidence, prevalence, mortality, YLD, YLL, and DALYs—for 23 age groups; males, females, and both sexes combined; and 204 countries and territories that were grouped into 21 regions and seven super regions.^1^ Details of the GBD 2019 study and the general process of the disease burden estimation are beyond the scope of this study. Herein, we focused on the methods and statistical analyses of estimation the stroke burden from the GBD 2019 study. The starting point of stroke estimates is cause‐specific mortality, which serves as the basis for estimating incidence, prevalence, YLD, YLL, and DALYs. Vital registration and verbal autopsy data were used to model stroke’s cause of death estimation. The International Classification of Diseases (ICD)‐10 codes were utilized for mapping to the GBD cause list^1^, ^14^, ^15^ (Supplementary Table 1), and were used as the input for a cause of death ensemble model (CODEm),^16^ to estimate cause‐specific mortality. The CODEm estimates trends in cause of death by investigating available data, such as education, lagged distributive income, cumulative cigarette smoking, and sociodemographic index. To ensure that the independently modeled single‐cause mortality estimates match the separately modeled all‐cause mortality, an algorithm called CoDCorrect^17^ was used, which scaled the single‐cause estimates into the all‐cause mortality envelope. Mortality‐to‐incidence ratios (MIR) were applied to the final stroke’s mortality measures to estimate the incidence. Incidence and survival were used to estimate prevalence. To estimate stroke YLL, each death caused by stroke was multiplied by the standard life expectancy at that age, which was calculated as 86.6 years at birth in the GBD 2016.^18^ YLD calculated by multiplying the prevalence of each sequela by the sequela‐specific disability weight. A brief measure of cause‐burden based on both nonfatal health losses and premature deaths was reported as DALYs, which were the addition of the YLL and YLD.

Risk factor burdens were estimated consistent with the general framework established for comparative risk assessment (CRA).^7^, ^19^, ^20^ Relative risk curves for each risk factor were estimated using spatiotemporal Gaussian process regression, DisMod‐MR 2.1, a Bayesian meta‐regression method, or alternative methods.^1^, ^7^ GBD study included 20 behavioral, environmental and occupational, and metabolic risk factors (alcohol use, ambient particulate matter pollution, diet high in red meat, diet high in sodium, diet low in fiber, diet low in fruits, diet low in vegetables, diet low in whole grains, high body mass index [BMI], high fasting plasma glucose [FPG], high LDL cholesterol, high systolic blood pressure [SBP], high temperature, household air pollution from solid fuels, kidney dysfunction, lead exposure, low physical activity, low temperature, secondhand smoke, and smoking) to estimate the attributable burden of stroke.^7^

### Sociodemographic indices

The Sociodemographic Index (SDI) is a composite indicator of development status strongly correlated with health outcomes. It is the geometric mean of 0 to 1 index of total fertility rate under the age of 25, mean education for those ages 15 and older, and lag‐distributed income per capita. As a composite, a location with an SDI of 0 would have a theoretical minimum level of development relevant to health, whereas a location with an SDI of 1 would have a theoretical maximum level.^21^ Each province was grouped based on SDI into 5 SDI quintiles, including low, low‐middle, middle, high‐middle, and high. Incidence, mortality, and stroke DALYs were compared between the SDI quintiles.

### Decomposition analysis

We calculated 2 scenarios to analyze the contribution of population aging, population growth, and changes in the age‐specific incidence rates on the absolute change of stroke incidence^22^; in the first scenario, the age structure, sex structure, and the age‐specific rates from 1990 were applied to the total population of the year 2019. The difference between the total number of cases in 1990 and the hypothetical scenario was attributed to population growth. In the second hypothetical scenario, the age‐specific rates from 1990 were applied to the age structure, sex structure, and population size of 2019. Differences between the second hypothetical scenario and the first hypothetical scenario were attributed to population aging. Differences between the total number of cases in 2019 and the second hypothetical scenario were attributed to changes in the age‐specific rates.

The GBD standard population was used to calculate the age‐standardized incidence rate (ASIR), age‐standardized death rate (ASDR), and age‐standardized DALY rate in this study. Data will be described in terms of absolute numbers and age‐standardized rates per 100,000 population following 95% uncertainty interval (UI), 25th and 975th values of the ordered draws. All the statistical analyses, plots, and numbers created in this study were performed by R for windows v 3.6.1 and RStudio v 1.0.136 (http://www.r‐project.org/, RRID: SCR_001905).^23^

## Results

### Prevalence and Incidence

**Figure 1:**
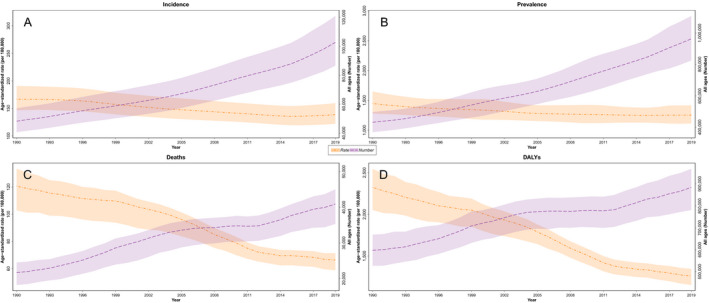
The time trend of all age numbers and age‐standardized rate of stroke. (A) Incidence, (B) Prevalence, (C) Deaths, and (D) DALYs. [Colour figure can be viewed at wileyonlinelibrary.com]

In 2019, the absolute number of strokes in Iran was 963,512 (95% UI: 859,232–1,079,662) (53.6% females). From that, 88.1% were ischemic, 12% were intracerebral hemorrhage, and 4.7% were subarachnoid hemorrhage. The stroke incident cases increased from 48,274 (42,265–55,623) in 1990 to 102,778 (90,115–117,821) in 2019, revealing a 2.1‐fold increase, whereas the ASIR of stroke decreased from 166.6 (146.6–190.9) in 1990 to 138.8 (121.8–159.6) per 100,000 population in 2019 (Fig. 1A). The ASIR of stroke had a 16.7% (−18.1 to −15.1) decrease in both genders (13.4% (−15.7 to −10.7) in females and 20.1% (−21.9 to −18.3) in males from 1990 to 2019 (Table 1). There was a decreasing trend of the incidence rate of stroke in all provinces in females and males, except for one province (Ilam), with an increase of 0.4% (−6.2 to 8.0) (Supplementary Table 2). At the subnational level, the highest and the lowest ASIR were 171.54 (148.87–201.32) (Golestan) and 116.88 (101.25–135.1) (Qom), respectively. The highest and lowest changes in the incidence rate of stroke among all provinces were −27.0% (−30.0 to −23.6) (Qom) and −4.2 (−8.3 to 0.0) (Ardebil), respectively.

**Table 1:** National age‐standardized rate of incidence, prevalence, deaths, DALYs, YLL, and YLD due to stroke in 1990 and 2019, with percentage change by sex.

Measure	Age‐standardized rate (per 100,000)						% Change (1990–2019)		
	1990			2019					
	Both	Female	Male	Both	Female	Male	Both	Female	Male
Incidence	166.6 (146.6 to 190.9)	166 (146.4 to 189.8)	167.8 (146.9 to 192.1)	138.8 (121.8 to 159.6)	143.7 (125.3 to 165.6)	134 (117.3 to 154.1)	−16.7 (−18.1 to −15.1)	−13.4 (−15.7 to −10.7)	−20.1 (−21.9 to −18.3)
Prevalence	1446.9 (1279.8 to 1649.5)	1590.6 (1412.9 to 1796.1)	1306.1 (1144.4 to 1502.8)	1253.8 (1113.5 to 1418.4)	1349.7 (1199.5 to 1520.2)	1159.3 (1022.9 to 1319.6)	−13.3 (−15.9 to −10.7)	−15.1 (−17.9 to −12.3)	−11.2 (−14.9 to −7.3)
Deaths	120.7 (102.8 to 133.5)	119.3 (97.9 to 132.8)	120.5 (99.8 to 136.2)	66.2 (58.7 to 71.3)	68.2 (59 to 74.4)	64.8 (57.3 to 70.3)	−45.1 (−50.6 to −35.4)	−42.8 (−49 to −28.2)	−46.3 (−53.9 to −37.9)
DALYs	2324.3 (2051.8 to 2547)	2273.2 (1968 to 2521.3)	2353.7 (2039.8 to 2680)	1262.2 (1153.5 to 1346.3)	1252.8 (1126.4 to 1360.2)	1274.9 (1173.4 to 1371.5)	−45.7 (−51 to −38.3)	−44.9 (−50.5 to −34.8)	−45.8 (−52.2 to −38.4)
YLLs	2099.4 (1838.1 to 2315.2)	2010.4 (1714.9 to 2235)	2165.6 (1872.3 to 2471.9)	1065.8 (976.7 to 1134.4)	1027.6 (914.9 to 1114.7)	1107.2 (1008.5 to 1192.1)	−49.2 (−54.5 to −41.4)	−48.9 (−54.7 to −38)	−48.9 (−55.5 to −41.1)
YLDs	225 (161.2 to 290.2)	262.8 (188.6 to 336.4)	188.1 (135 to 244.4)	196.4 (140.3 to 251.9)	225.2 (162.4 to 288.2)	167.7 (119.6 to 216.7)	−12.7 (−15.5 to −10)	−14.3 (−17.2 to −11.4)	−10.9 (−14.6 to −6.8)

**Figure 2:**
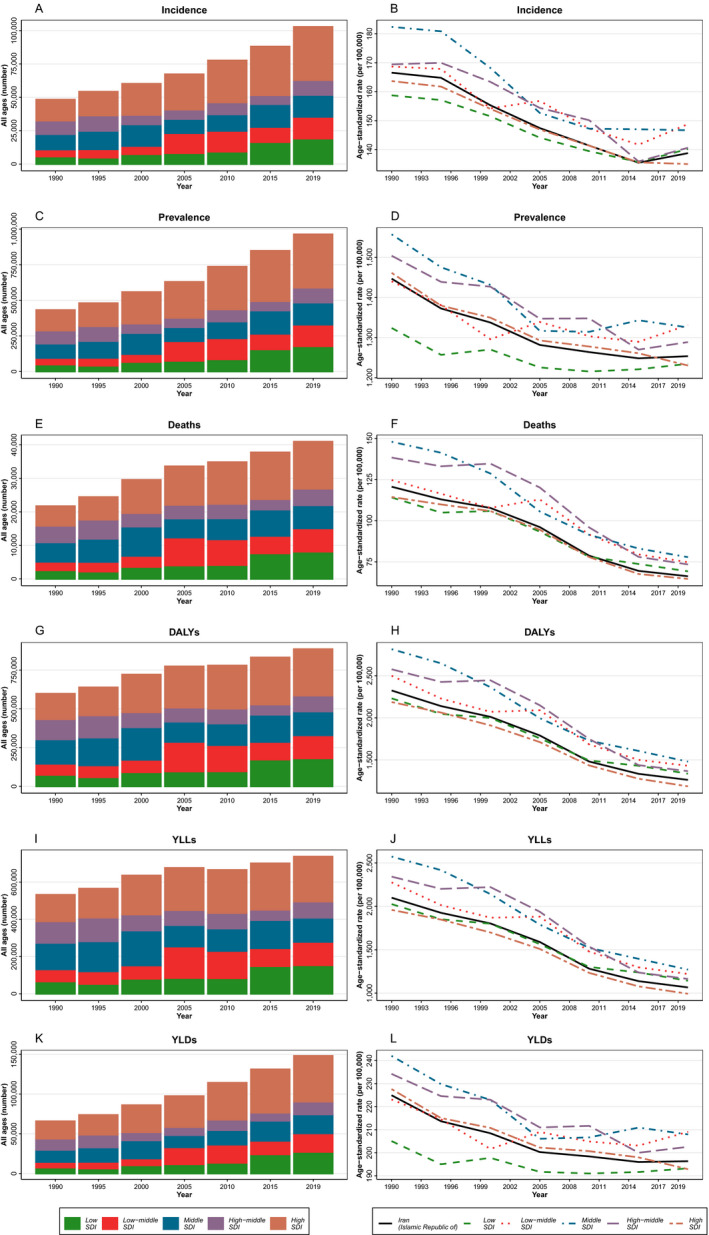
The time trend of all age numbers and age‐standardized rate of stroke. (A, B) Incidence, (C, D) Prevalence, (E, F) Deaths, (G, H) DALYs, (I, J) YLL, and (K, L) YLD categorized by 5 SDI quintiles. [Colour figure can be viewed at wileyonlinelibrary.com]

Although stroke incident cases showed an increasing pattern, ASIR decreased in all SDI quintiles between 1990 and 2019 and the decrease was more prominent in middle SDI (182.38 in 1990 to 146.70 in 2019) and high‐middle SDI (169.44 in 1990 to 140.65 in 2019) provinces. ASIR was highest in the low‐middle SDI quintile (148.80) and lowest in the high SDI quintile (135.02) in 2019. However, high SDI quintiles had the highest number of incident cases during the follow‐up period. The ASIR at the national level had a decreasing pattern, except for the last 4 years. In comparison with ASIR in Iran, low‐middle, middle, and high‐middle SDI quintiles had higher ASIR between 1990 and 2019 (Fig. 2A).

**Figure 3:**
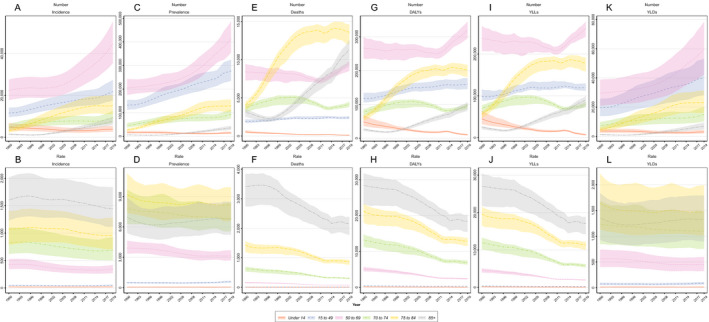
The time trend of number and rate of stroke (A, B) Incidence, (C, D) Prevalence, (E, F) Deaths, (G, H) DALYs, (I, J) YLL, and (K, L) YLD categorized by age groups. [Colour figure can be viewed at wileyonlinelibrary.com]

With regard to the age groups, the highest and lowest incidence rates were 1449.59 (1113.72–1842.66) (85 years plus age group) and 18.84 (12.84–26.71) (under 14 years age group). The incidence rate of stroke declined in all age groups during the follow‐up period. The number of incident cases was higher in the age group of 50–69 years and had the biggest increase among all age groups from 2005 to 2019 (Fig. 3A).

Decomposition analysis of new cases at the national level showed an overall 112.9% increase from 1990 to 2019, in which incidence rate change was responsible for a 46.9% decrease in this ratio; while 115.8% and 44% of the changes in incidence were attributable to age structure change and population growth, respectively. This pattern was observed in both sexes and in all provinces (Supplementary Table 3, Table 2).

**Table 2:** Decomposition analysis of stroke new cases between 1990 and 2019 at national and subnational levels.

Location		New cases		Expected new cases in 2019		% 1990–2019 new cases change cause			% 1990–2019 new cases overall change
		1990	2019	Population growth	Population growth + Aging	Population growth	Age structure change	Incidence rate change	
Iran (Islamic Republic of)		48274	102778	69515	125436	44%	115.8%	−46.9%	112.9%
Subnational	Alborz	1147	3640	2241	4523	95.4%	199%	−77%	217.4%
	Ardebil	907	1810	1005	1920	10.9%	100.9%	−12.2%	99.6%
	Bushehr	581	1398	996	1690	71.4%	119.6%	−50.2%	140.8%
	Chahar Mahaal and Bakhtiari	537	1145	727	1338	35.3%	113.8%	−35.8%	113.4%
	East Azarbayejan	3271	5731	3865	7213	18.2%	102.4%	−45.3%	75.2%
	Fars	2889	6393	3928	7455	35.9%	122.1%	−36.8%	121.3%
	Gilan	2406	4726	2685	5560	11.6%	119.5%	−34.7%	96.4%
	Golestan	1190	2672	1713	3008	43.9%	108.8%	−28.3%	124.4%
	Hamadan	1500	2642	1551	3037	3.4%	99.1%	−26.3%	76.2%
	Hormozgan	772	1814	1583	2234	105%	84.3%	−54.4%	135%
	Ilam	276	621	364	695	32%	119.9%	−26.9%	125%
	Isfahan	2964	6031	4064	7779	37.1%	125.3%	−59%	103.4%
	Kerman	1351	3157	2409	3854	78.3%	107%	−51.6%	133.7%
	Kermanshah	1510	2853	1757	3485	16.3%	114.5%	−41.8%	89%
	Khorasan‐e‐Razavi	4435	7615	6270	10363	41.4%	92.3%	−62%	71.7%
	Khuzestan	2520	5988	3835	6562	52.2%	108.2%	−22.8%	137.6%
	Kohgiluyeh and Boyer‐Ahmad	324	752	492	873	51.9%	117.6%	−37.2%	132.3%
	Kurdistan	1013	2009	1356	2444	33.8%	107.4%	−42.9%	98.3%
	Lorestan	1220	2239	1395	2706	14.3%	107.4%	−38.3%	83.5%
	Markazi	1165	2106	1389	2685	19.2%	111.3%	−49.7%	80.8%
	Mazandaran	2368	5441	3113	6543	31.5%	144.9%	−46.5%	129.8%
	North Khorasan	563	1116	774	1350	37.5%	102.5%	−41.7%	98.2%
	Qazvin	735	1598	1028	1842	39.9%	110.8%	−33.2%	117.5%
	Qom	543	1258	999	1752	84.1%	138.7%	−91.1%	131.7%
	Semnan	526	978	815	1267	54.9%	85.9%	−55%	85.8%
	Sistan and Baluchistan	994	2189	2007	2438	101.9%	43.4%	−25%	120.3%
	South Khorasan	612	978	761	1178	24.2%	68.1%	−32.5%	59.8%
	Tehran	6668	17078	10924	20695	63.8%	146.5%	−54.2%	156.1%
	West Azarbayejan	1924	4080	2821	4892	46.6%	107.7%	−42.3%	112.1%
	Yazd	602	1299	1019	1589	69.2%	94.6%	−48.2%	115.5%
	Zanjan	762	1421	926	1722	21.6%	104.5%	−39.5%	86.5%

### Mortality

The number of stroke deaths in Iran increased from 21,698 (18,964–23,908) in 1990 to 40,912 (36,741–43,848) in 2019. Moreover, the ASDR of stroke decreased from 120.7 (102.8–133.5) in 1990 to 66.2 (58.7–71.3) in 2019 and showed a 45.1% (50.6–35.4) decrease (Fig. 1B, Table 1).

The ASDRs in females and males were 68.2 (59.0–74.4) and 64.8 (57.3–70.3) per 100,000 population, respectively. Males had a slightly higher decline in ASDR between 1990 and 2019 compared with women (Males: −46.3% [−53.9 to −37.9], Females: −42.8 [−49.0 to −28.2]) (Table 1).

The number of deaths increased and the ASDR decreased in all SDI quintiles between 1990 and 2019. Despite the decreasing pattern of ASDR in low–middle SDI provinces, there was a peak in both ASDR and death numbers in 2005. Middle and high‐middle SDI quintiles had the largest decrease in ASDR between 1990 and 2019. High SDI provinces had the highest number of deaths and the lowest ASDR (Fig. 2C).

There were significantly big rises in the number of deaths in the age groups of 75–84 years and 85 plus. The death rate in the age group of 85 plus was widely higher compared to other age groups with a decreasing pattern **(**Fig. 3C **).**

The pattern of ASDR at the subnational level was similar to the national trend. The highest ASDR was observed in Bushehr (97.0 [81.0–107.6]/100,000 population) and the lowest was observed in Tehran (37.8 [31.4–44.4]/100,000 population). The highest and lowest changes in the death rate of stroke among all provinces were −59.0% (−68.0 to −45.5) (Qom) and −28.6% (−41.9 to −9.2) (Ilam), respectively (Supplementary Table 2).

**Figure 4:**
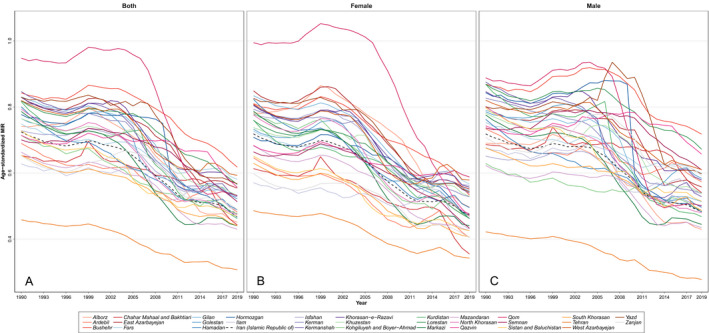
The time trend of national and subnational trends in stroke age‐standardized MIR by sex (A. both, B. Female, and C. Male). [Colour figure can be viewed at wileyonlinelibrary.com]

The findings revealed that the age‐standardized MIR as a proxy for quality of care at the national level continuously declined from 0.72 in 1990 to 0.48 in 2019. The pattern was similar for all provinces except for one (Qom) in which the age‐standardized MIR was near 1 in both genders and above 1 in females between 1996 and 2007. There was a province that always had the lowest age‐standardized MIR with decreasing pattern (Tehran) (Fig. 4).

### YLL, YLD, DALYs

Stroke was responsible for 4.48% (3.95% to 4.99%) of total DALYs in 2019, including 3.38% (2.97% to 3.77%) due to ischemic stroke, 0.87% (0.76% to 0.98%) due to intracerebral hemorrhage, and 0.22% (0.19% to 0.26%) due to subarachnoid hemorrhage. The number of stroke DALYs increased from 597,142 (547,356 to 651,017) in 1990 to 884,768 (812,248 to 943,655) in 2019. Although the age‐standardized DALYs rate of stroke per 100,000 population declined about 45.7% (−51.0 to −38.3) from 2324.3 (2051.8 to 2547.0) in 1990 to 1262.2 (1153.5 to 1346.3) in 2019. A similar pattern was observed in both males and females (Table 1, Fig. 1D).

The age‐standardized DALYs rate showed a decreasing pattern in all SDI quintiles between 1990 and 2019. Although the age‐standardized YLL rate showed decreasing trends similar to the age‐standardized DALYs rate, the age‐standardized YLD rate was almost steady from 2005 to 2019 (Fig. 2).

With regard to the age groups, the number of DALYs and YLL were highest in the population of 50–69 years, but the rate of DALYs and YLL were highest in the age group of 85 plus. Moreover, the age group of 50–69 years had the highest number of YLD with an increasing pattern (Fig. 3).

The pattern of the age‐standardized DALYs rates at subnational levels was similar to the national level. The highest and lowest changes in DALYs rates were observed in Qom with a 58.1% (−67.0 to −45.9) decline and in Ilam with a 32.3% (−44.2 to −15.3) decline, respectively (Supplementary Table 2).

The age‐standardized YLL and YLD rates of stroke per 100,000 population have decreased from 2099.4 (1838.1 to 2315.2) in 1990 to 1065.8 (976.7 to 1134.4) in 2019 and from 225.0 (161.2 to 290.2) in 1990 to 196.4 (140.3 to 251.9) in 2019, respectively **(**Table 1 **).**

**Figure 5:**
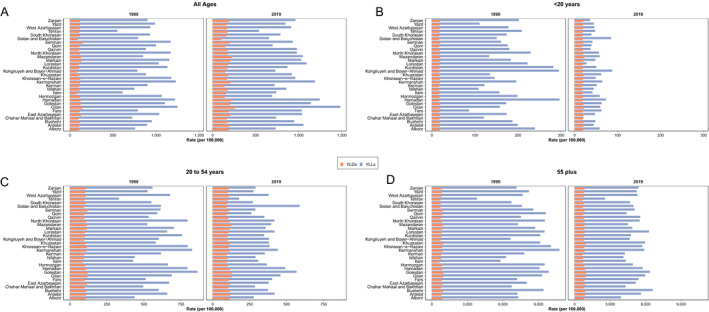
DALYs rate due to stroke by share of YLD and YLL and age group at subnational level (A. all ages, B. <20 years, C. 20–54 years, and D. 55+). [Colour figure can be viewed at wileyonlinelibrary.com]

The changes in the rate of YLL and YLD in different provinces from 1990 to 2019 by age groups are shown in Figure 5. In the age group of <20 years, the rate of YLL significantly decreased in all provinces. However, there were no significant changes in the rate of YLD during this period. In the age group of 20 to 54 years, the rate of YLL decreased in most provinces, except for Sistan and Blauchestan. The rate of YLD also did not show a significant decrease during this time. In the age group of >55 years, the rates of YLL and YLD were significantly higher than other age groups. Indeed, in this group, the rate of YLL decreased and the rate of YLD remained unchanged during the follow‐up period (Fig. 5).

**Figure 6:**
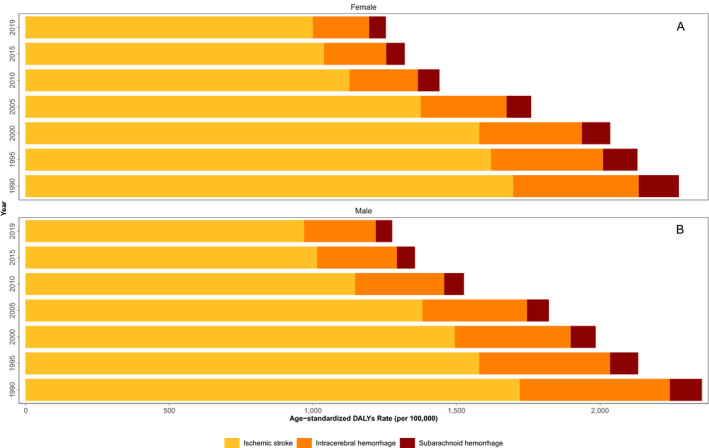
The trend of stroke age‐standardized DALYs rate by sex and pathological subtypes (A. female and B. male). [Colour figure can be viewed at wileyonlinelibrary.com]

The trend of age‐standardized DALYs rate of three subtypes of stroke (ischemic stroke, intracerebral hemorrhage, and subarachnoid hemorrhage) is shown in Figure 6. In both genders, DALYs rates of all three subtypes of stroke declined from 1990 to 2019 and the large proportion of DALYs rates were caused by ischemic stroke and intracerebral hemorrhage.

### Stroke burden attributable to risk factors

In 2019, the stroke ASDR and DALYs attributable to all risk factors at the national level were 54.7 (47.4 to 61.1) and 1072.1 (972.6 to 1165.2) per 100,000 population and had a 43.6% (−49.3 to −33.2) and 43.9% (−49.4 to −35.4) decline during the follow‐up period, respectively. This pattern was also observed in all provinces and in both sexes (Supplementary Table 4).

**Figure 7:**
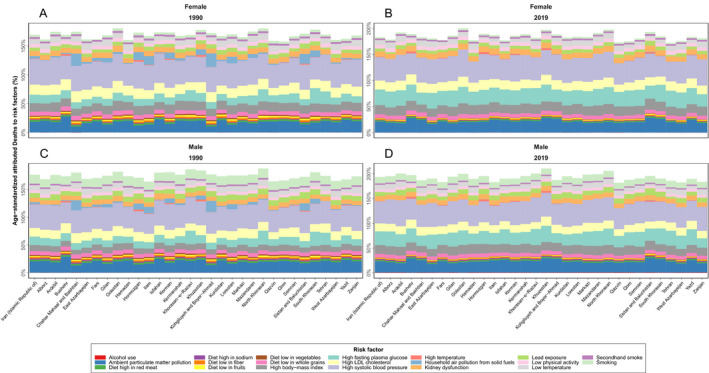
National and subnational ASDR attributed to risk factors by sex in 1990 (A. female and C. male) and 2019 (B. female and D. male). [Colour figure can be viewed at wileyonlinelibrary.com]

**Figure 8:**
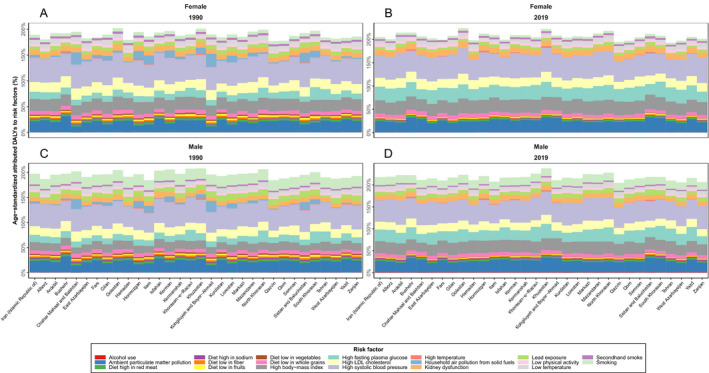
National and subnational age‐standardized DALY rates attributed to risk factors by sex in 1990 (A. female and C. male) and 2019 (B. female and D. male). [Colour figure can be viewed at wileyonlinelibrary.com]

Of national stroke ASDRs in 2019, 44.7% (35.7 to 54.7%) were attributable to high SBP, 28.8% (15.2 to 57.4) to high FPG, 21.9% (19.2 to 24.8) to ambient particulate matter pollution, 17.8% (11.4 to 24.4) to high BMI and 16.6% (6.1 to 33.9) to high LDL cholesterol. Between 1990 and 2019, stroke ASDRs attributable to alcohol use and high FPG increased (251.4% and 5.1%), whereas ASDRs attributable to other 18 related risk factors were decreased as household air pollution from solid fuels (−99.3%), and diet low in vegetables (−80.3%) had the most decrease among them. The stroke ASDR attributed to smoking was 5.2 (4.6 to 5.9) per 100,000 population in Iran and was higher in males (8.5 per 100,000 population) compared with females (2.0 per 100,000 population) in all provinces. The age‐standardized attributed DALYs rate in 2019 were mostly attributed to high SBP (612.9 [506.3 to 718.1]), high BMI (353.2 [248.5 to 462.5]), high FPG (353.2 [207.7 to 596.8]) and ambient particulate matter pollution (325.0 [280.7 to 372.3]) per 100,000 population. The age‐standardized rate of DALYs attributed to high LDL cholesterol and smoking were 234.3 (135.1 to 399.7) and 145.3 (129.3 to 161.8) per 100,000 population (Figs. 7, 8).

## Discussion

In this study, we used the GBD 2019 study results to analyze the national and subnational incidence and burden of stroke in Iran. We showed that the incidence rate of stroke is decreasing nationally and subnationally except for the last 4 years, during which the ASIR was slightly increasing in low‐, low‐middle, and high‐middle SDI quintiles; however, the number of incident cases are increasing in all SDI quintiles. Total death number of strokes increased over the study period possibly due to population growth, and ASDR decreased at national and subnational levels and in all SDI quintiles. We also showed that ASIR and ASDR were slightly higher in women compared with men in 2019. Moreover, we showed that the number of new cases and DALYs is increasing among the young population (15–69 years).

Population aging,^24^ overall population growth, and reduced case fatality due to stroke are the probable causes of the increase in the number of incidents and prevalent cases of stroke. According to the increase in the incidence of stroke in young adults, stroke is no longer considered as the disease of old age. Changes in behavioral risk factors, such as smoking,^25^ alcohol consumption,^26^ physical activity,^27^ and dietary factors,^28^ are likely to account for the increase in incident cases and also ASIR in low‐, low‐middle, and high‐middle SDI quintiles during the past 4 years. Similar to our findings, the absolute number of stroke incident cases and deaths had an increasing pattern all over the world; however, this increase was significantly higher in Asia and especially in middle‐income countries (South‐Asian countries such as India, Pakistan, and Bangladesh, and in developing countries in South‐East Asia, such as Cambodia, Indonesia, and Malaysia).^29^, ^30^ However, similar to Iran, there was a reduction in ASIR in Japan, Singapore, and Korea between 1990 and 2010.^31^ Moreover, stroke‐related mortality has been decreasing in East‐Asian countries such as Japan, Korea, Taiwan, and the urbanized areas of China.^32^ In the European Union, the ASIR [211.7 (204–219)] and ASDR [85 (80.7–89.6)] in 2017, was higher compared with 2019 rates in Iran.^33^ During the past 2 decades, there was a 23.8% (15.9–29.3) reduction of stroke ASDR in the MENA region, moreover, ASIR and DALYs reduced 6.0% (2.9–9.0) and 26.8% (20.8–31.3), respectively^34^; however, in this study, we reported more reduction of ASDR, ASIR, and DALYs in Iran. Similar to our findings, results from the NASBOD study^11^ in Iran, showed an increasing trend in absolute number of strokes and their related deaths and a decreasing pattern in stroke ASDR between 1990 and 2015.

The decline in ASDR is probably due to advances in treatment methods (such as thrombectomy)^35^ and acute management (such as early thrombolysis treatment)^36^ during past years. However, this decline may be partly explained by the reduction the incidence rate of stroke, and it is difficult to determine which one had the greater effect on the mortality rate. Moreover, the inequality in ASIR and ASDR in women compared with men may be explained by longer life span, higher frequency of stroke risk factors, hormonal status, and risks related to pregnancy in women.^37^

The overall burden of stroke, as measured by age‐standardized DALYs rates, showed a decreasing pattern in all SDI quintiles; however, the absolute number of DALYs increased between 1990 and 2019. This increase in DALYs numbers may be due to population growth and aging, better stroke survival, and thus higher prevalence of chronic stroke cases. In addition, the increase in DALYs number in the young population may be due to a rise in cardiovascular risk factors among younger adults in low‐ and middle‐income countries.^38^

The burden of stroke was shown to be attributed to modifiable risk factors such as high SBP, BMI, FPG, and LDL. Targeting these risk factors and applying appropriate screening methods may be effective for the prevention of stroke. According to our results, a great proportion of national stroke ASDRs were attributable to high SBP and high FPG levels. Although stroke ASDRs attributable to all risk factors had a decreasing pattern between 1990 and 2019, ASDRs attributable to alcohol use and high FPG increased.

The association between HTN and stroke is due to the strain that high blood pressure puts on vessels and leads to atherosclerosis (ischemic stroke) or bursting the blood vessels inside the brain (hemorrhagic stroke).^39^ Furthermore, consumption of salt among the Iranian population is higher than the recommended levels and high salt intake has been shown to be associated with increased risk of stroke, through the effect on SBP.^40^ Studies reported that high SBP had a high mortality impact among the Iranian population, despite the program for controlling hypertension in high‐risk patients.^41^, ^42^ The awareness and control of hypertension is poor in Iran and with applying more strict cut‐offs, a higher proportion of adults (mostly aged 25–34 years) will be in the hypertensive category.^43^ Therefore, a comprehensive program in all provinces and regions in Iran including both high‐risk and low‐risk populations is necessary to reduce the risk of stroke. Moreover, treatment with blood pressure medications^44^ may be useful in the prevention of stroke.

Obesity is associated with several conditions, such as HTN, impaired FPG, and high cholesterol, which may cause atherosclerosis and increase the risk of stroke.^45^ Diabetes and prediabetes are known to be associated with increased risk of stroke in all age groups; especially in young adults,^46^, ^47^ due to endothelial dysfunction, vascular stiffness, and systemic inflammation.^48^

Excessive LDL cholesterol levels may build up fatty deposits on artery walls and leads to narrow, atherosclerotic arteries and thus, increase the risk of ischemic stroke.^49^ There is a high prevalence of dyslipidemia among the Iranian adult population, probably due to a sedentary lifestyle and high‐fat diets.^50^ Indeed, many studies showed that treatment with lipid‐lowering agents^51^, ^52^ may lead to cost‐effective primary and secondary stroke prevention.

Our data showed that the overall burden of stroke in the middle‐aged population (50–69 years) is increasing, with significant increases in the absolute number of incident cases and DALYs. There was also a significant increase in the absolute number of young adults (15–49 years) living with stroke. This increase may be due to the high prevalence of cardiovascular risk factors (such as alcohol and cigarette smoking),^53^, ^54^ high prevalence of obesity, and changes in awareness of stroke symptoms and more refers to specialists among the young population. Moreover, there are several reports that showed the increasing pattern of stroke incidence in the young population.^55^, ^56^ Therefore, considering preventive plans are important to reduce the burden of stroke in this age group.

Improvement of stroke care services plays a beneficial role in stroke prevention and its morbidity reduction.^57^ In Iran, supplementing training of nurses and paramedics, supplying each ambulance with general practitioners, nurses, and paramedics,^58^ and modification of NIH Stroke Scale/Score to simpler the stroke severity assessment^59^ helped to reach our goals. Stroke is preventable by modifying risk factors; thus, applying preventive measures, especially for the young population, in addition to improving acute stroke care services would be beneficial to reduce the risk of stroke. Other strategies that would improve stroke prevention include the development of risk factors screening and treatment programs, delivery of primary healthcare to control risk factors, providing stroke prevention medicines, and development of school health programs to educate and modify lifestyle from an early age.

### Strengths and Limitations

This study has many strengths such as systematic data and method usage which could potentially compare the risk of stroke among different provenances and years, possibly making the results generalizable. To the best of our knowledge, there were no lack of data from the epidemiologic indices of stroke in all age and sex groups and provincial levels throughout the study. Furthermore, we reported modest reliable data from the valuable GBD study, which is a large‐scaled and well‐known epidemiological study.

There are some known limitations for epidemiological studies such as the accuracy of the estimates of ASDR of stroke was limited by the accuracy and availability of provenance’s epidemiologic data. There was a lack of adequate data to estimate first‐ever and recurrent stroke in different provenances, which would not allow us to provide a more complete aspect of stroke burden and adequately estimate the success of previous strategies.

## Conclusion

Our study provided a national and subnational burden of stroke across all distributions of sex and age during 29 years in Iran. In 1990, Iran was placed at the 13th rank of stroke ASDR in the MENA region, hopefully in 2019, Iran was placed at the 16th position. According to our study, ASDR for stroke has decreased in the past 29 years. Hence, the new cases of stroke and the stroke death number have increased during these years mostly due to population growth. Reported findings in this study could be used to identify which population and region are in jeopardy. Furthermore, it is beneficial to identify the most menacing risk factors for stroke and justify the preventive strategies in the future.

### Role of the funding source

The funders of this study had no role in study design, data collection, data analysis, data interpretation, or writing of the report. The corresponding author had full access to all the data in the study and had final responsibility for the decision to submit for publication.

## Ethics approval

This study was approved by the ethical committee of Endocrinology and Metabolism Research Institute of Tehran University of Medical Sciences, under code IR.TUMS.EMRI.REC.1400.023.

## Conflict of Interest

The author(s) declared no potential conflict of interest with respect to the research, authorship, and/or publication of this article.

## Author Contributions

AF, ZE, and AS contributed to writing the manuscript and suggested statistical methods for analyzing the data. SSM participated in data acquisition, conducted data analyses, and interpreted results. MK, YST, NR, EG, SA, EM, SK, SS, NR, and BL participated in data acquisition and critical review of the manuscript. FF conceived the research questions and provided guidance on data acquisition and analysis and interpreting results and critical review of the manuscript. All authors read and approved the final manuscript.

## Supporting information


**Supplementary Figure S1** The Global Burden of Disease (GBD) 2019 cause of death, mortality, and years of life lost (YLLs) estimation flow chart.

**Supplementary Table S1** List of ICD‐10 mapped codes for stroke.

**Supplementary Table S2** Subnational age‐standardized rate of incidence, prevalence, deaths, DALYs, YLLs, and YLDs due to stroke in 1990 and 2019, with percentage change by sex.

**Supplementary Table S3** Decomposition analysis of stroke new cases between 1990 and 2019 by sex at national and subnational levels.

**Supplementary Table S4** National and subnational age‐standardized rate of attributed deaths, DALYs, YLLs, and YLDs due to all risk factors in 1990 and 2019, with percentage change by sex.

## Data Availability

The data set(s) supporting the conclusions of this article is available on request.
