# Supplementary material for: National and subnational burden of stroke in Iran from 1990 to 2019
Source: Ann Clin Transl Neurol. 2022 Apr 8;9(5):669–83. doi: 10.1002/acn3.51547 (PMC9082377; doi:10.1002/acn3.51547)

Supplementary figure 1. The Global Burden of Disease (GBD) 2019 cause of death, mortality, and years of life lost (YLLs) estimation flow chart

Stroke

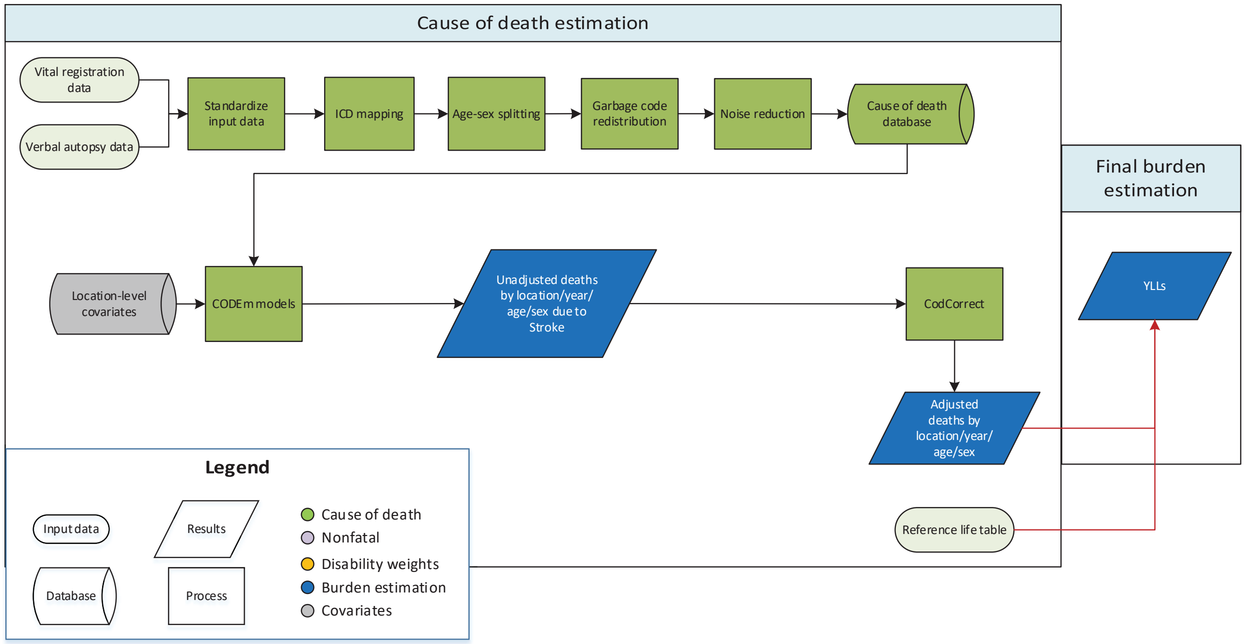

Ischaemic Stroke

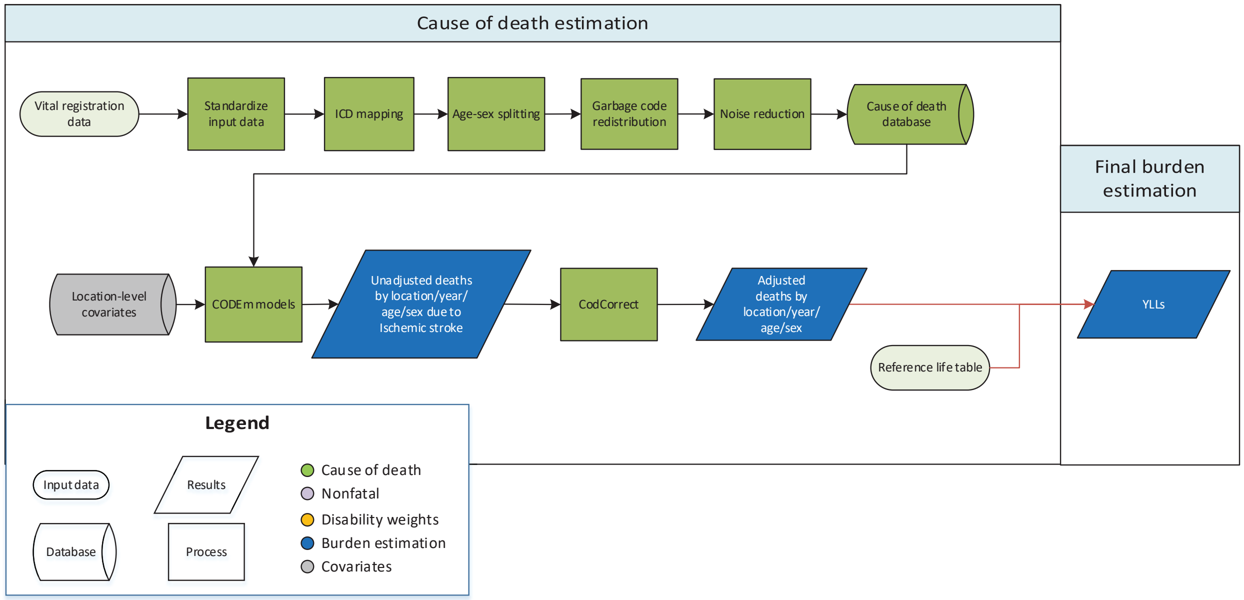

Intracerebral haemorrhage

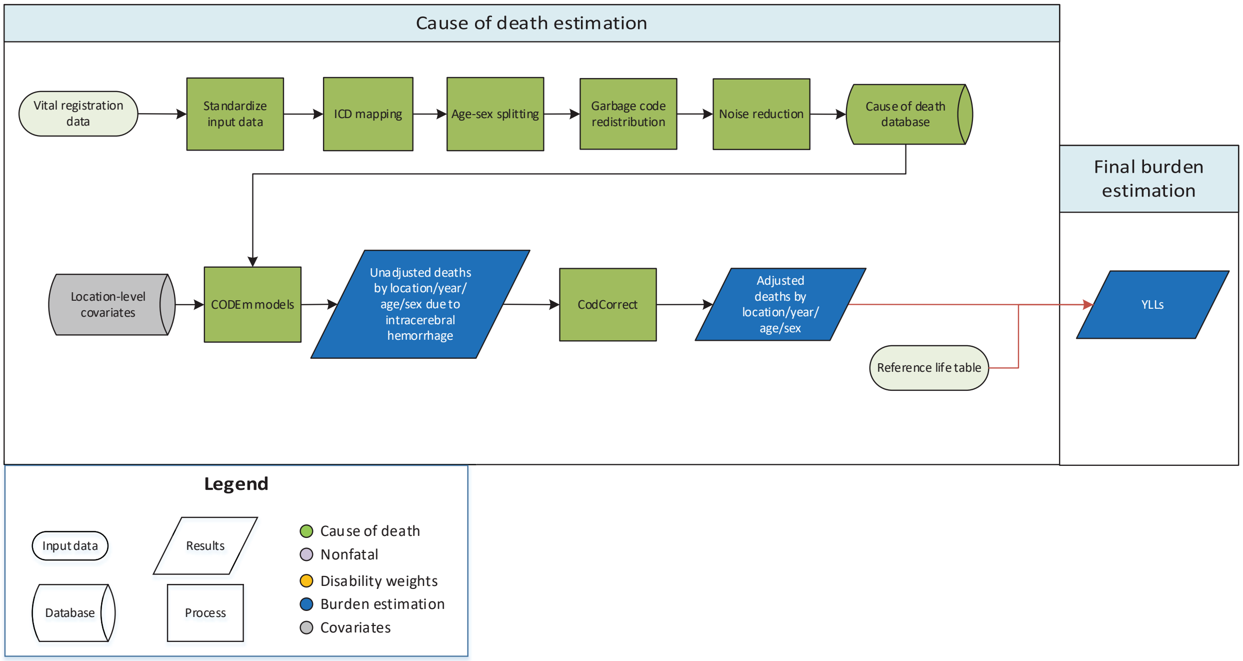

Subarachnoid haemorrhage

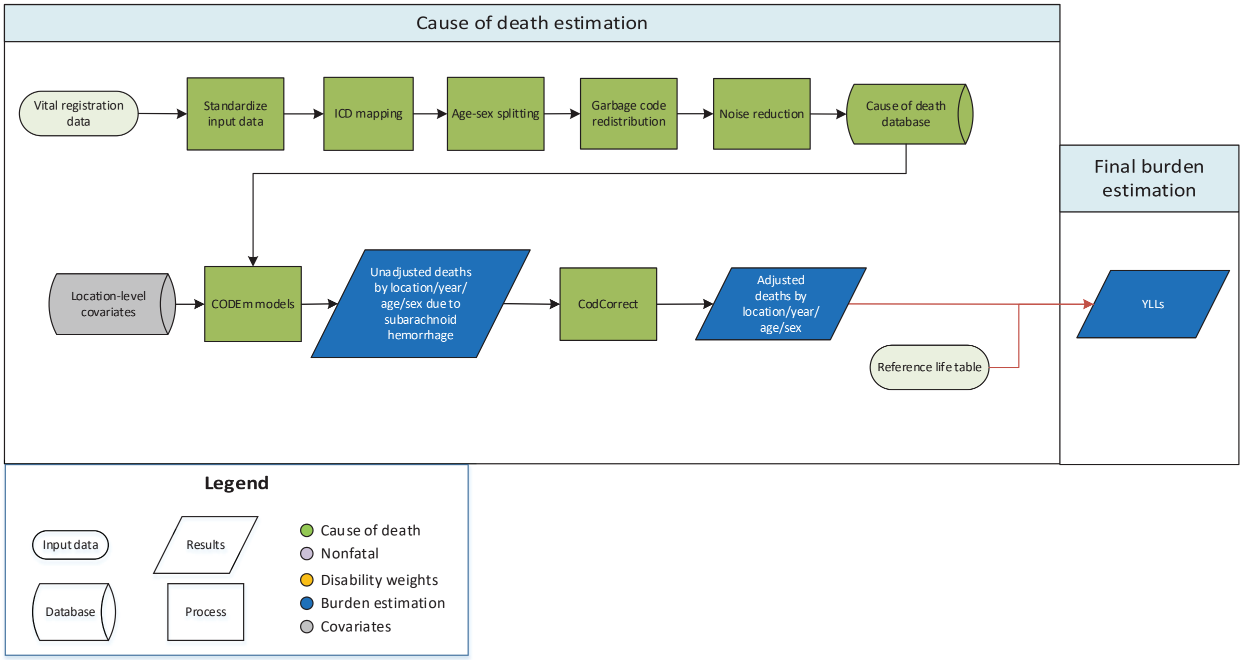

Supplement: Supplementary file 1 — Supplementary Figure S1 The Global Burden of Disease (GBD) 2019 cause of death, mortality, and years of life lost (YLLs) estimation flow chart. [file ACN3-9-669-s002.pdf]
