# Supplementary material for: National and subnational burden of stroke in Iran from 1990 to 2019
Source: Ann Clin Transl Neurol. 2022 Apr 8;9(5):669–83. doi: 10.1002/acn3.51547 (PMC9082377; doi:10.1002/acn3.51547)
Supplement: Supplementary file 2 — Supplementary Table S1 List of ICD‐10 mapped codes for stroke. [file ACN3-9-669-s004.pdf]

Supplementary Table 1. List of ICD-10 mapped codes for stroke

Measure	Cause	ICD-10
New cases (9)	Ischemic stroke	G45-G46.8, I63-I63.9, I65-I66.9, I67.2-I67.848, I69.3-I69.4
	Intracerebral hemorrhage	I61-I62, I62.9, I69.0-I69.298
	Subarachnoid hemorrhage	I60-I60.9, I67.0-I67.1
Deaths (1)	Ischemic stroke	G45-G46.8, I63-I63.9, I65-I66.9, I67.2-I67.3, I67.5-I67.6, I69.3
	Intracerebral hemorrhage	I61-I62, I62.1-I62.9, I68.1-I68.2, I69.1-I69.2
	Subarachnoid hemorrhage	I60-I60.9, I62.0, I67.0-I67.1, I69.0
