# Supplementary material for: National and subnational burden of stroke in Iran from 1990 to 2019
Source: Ann Clin Transl Neurol. 2022 Apr 8;9(5):669–83. doi: 10.1002/acn3.51547 (PMC9082377; doi:10.1002/acn3.51547)
Supplement: Supplementary file 3 — Supplementary Table S2 Subnational age‐standardized rate of incidence, prevalence, deaths, DALYs, YLLs, and YLDs due to stroke in 1990 and 2019, with percentage change by sex. [file ACN3-9-669-s005.pdf]

Supplementary Table 2. Sub-national age-standardized rate of incidence, prevalence, deaths, DALYs, YLLs, and YLDs due to stroke in 1990 and 2019, with percentage change by sex

Province	Measure	Age-standardized rate (per 100,000)						% Change (1990 to 2019)		
		1990			2019					
		Both	Female	Male	Both	Female	Male	Both	Female	Male
Alborz	Incidence	173.54 (152.3 to 200.4)	178.28 (155.65 to 206.81)	169.71 (147.98 to 196.19)	143.34 (124.57 to 166.56)	150.02 (129.92 to 174.91)	138.03 (119.42 to 159.9)	-17.4 (-20.5 to -14)	-15.8 (-21 to -10.3)	-18.7 (-23.4 to -13.3)
	Prevalence	1547.49 (1358.33 to 1753.6)	1743.19 (1531.78 to 1979.74)	1368 (1183.13 to 1576.29)	1330.95 (1165.82 to 1515.34)	1452.44 (1274.81 to 1659.47)	1220.58 (1055.8 to 1399.2)	-14 (-19 to -8.9)	-16.7 (-23.4 to -8.9)	-10.8 (-18.5 to -3.2)
	Deaths	128.64 (100.59 to 157.15)	139.51 (104.31 to 174.07)	117.21 (83.83 to 156.15)	64.61 (55.37 to 74.32)	74.51 (61.42 to 88.23)	59.22 (47.39 to 71.91)	-49.8 (-61.2 to -33.8)	-46.6 (-60.2 to -23.8)	-49.5 (-64 to -28.3)
	DALYs	2323.79 (1859.77 to 2866.29)	2467.5 (1872.74 to 3087.25)	2172.3 (1572.92 to 2853.6)	1192.59 (1047.54 to 1355.97)	1266.34 (1071.49 to 1465.47)	1154.81 (954.39 to 1379.7)	-48.7 (-59.9 to -31.7)	-48.7 (-61.5 to -28.6)	-46.8 (-61.8 to -25.4)
	YLLs	2081.08 (1615.07 to 2625.24)	2177.13 (1557.18 to 2801.23)	1973.77 (1381.79 to 2649.48)	983.66 (851.4 to 1130.68)	1022.68 (849.48 to 1210.13)	977.89 (791.52 to 1193.89)	-52.7 (-64.2 to -35.2)	-53 (-65.9 to -30.6)	-50.5 (-65.5 to -27.8)
	YLDs	242.71 (175.24 to 315.46)	290.37 (210.57 to 375.39)	198.53 (140.2 to 260.45)	208.93 (149.54 to 270.3)	243.66 (173.96 to 317.2)	176.93 (125.37 to 230.62)	-13.9 (-19.4 to -8.2)	-16.1 (-23.9 to -7.3)	-10.9 (-18.9 to -2.2)
Ardebil	Incidence	162.03 (141.29 to 185.76)	162.79 (141.69 to 187.58)	161.34 (140.37 to 185.05)	155.28 (134.93 to 179.05)	161.85 (140.51 to 187.97)	148.2 (128.27 to 170.44)	-4.2 (-8.3 to 0)	-0.6 (-6.5 to 5.2)	-8.1 (-14 to -1.7)
	Prevalence	1425.01 (1243.44 to 1615.43)	1569.79 (1376.84 to 1773.21)	1290.42 (1111.42 to 1497.35)	1360.33 (1200.93 to 1539.8)	1466.71 (1299.45 to 1654.01)	1250.41 (1089.33 to 1425.26)	-4.5 (-10.4 to 1.7)	-6.6 (-13.5 to 1.2)	-3.1 (-11.7 to 6.6)
	Deaths	111.78 (87.93 to 136.94)	105.2 (79.38 to 130.41)	118.43 (88.94 to 152.37)	74.84 (65.17 to 83.58)	65.45 (55.38 to 75.06)	84.95 (71.78 to 96.58)	-33 (-47.5 to -13.5)	-37.8 (-52.8 to -13.5)	-28.3 (-46.5 to -5)
	DALYs	2289.64 (1903.92 to 2750.95)	2202.51 (1755.49 to 2765.79)	2368.22 (1861.14 to 3016.63)	1456.54 (1293.08 to 1618.51)	1285.12 (1111.63 to 1463.74)	1637.6 (1401.49 to 1864.88)	-36.4 (-49 to -21.5)	-41.7 (-54.9 to -23.8)	-30.9 (-48.7 to -9.5)
	YLLs	2069.96 (1680.85 to 2543.16)	1944.69 (1493.15 to 2480.28)	2182.94 (1670.06 to 2837.45)	1243.61 (1096.71 to 1393.19)	1040.68 (892.86 to 1202.13)	1457.42 (1229.42 to 1670.81)	-39.9 (-53.1 to -23.8)	-46.5 (-60.8 to -26.7)	-33.2 (-51.6 to -10.2)
	[illegible]	219.68 [illegible]	257.82 [illegible]	185.28 [illegible]	212.93 [illegible]	244.44 [illegible]	180.18 [illegible]	-3.1 (-9.6 to [illegible]	-5.2 (-13 to [illegible]	-2.8 (-12.6 to [illegible]

		284.77)	333.27)	238.58)	275.64)	315.65)	233.71)	4.1)	3.7)	8.4)

Province	Measure	Age-standardized rate (per 100,000)						% Change (1990 to 2019)		
		1990			2019					
		Both	Female	Male	Both	Female	Male	Both	Female	Male
Bushehr	Incidence	187.13 (163.23 to 218.77)	182.92 (158.95 to 213.34)	192.05 (165.94 to 225.19)	157.03 (135.99 to 183.25)	167.27 (144.85 to 195.67)	146.62 (125.52 to 172.22)	-16.1 (-19.7 to -12.1)	-8.6 (-14.6 to -2.3)	-23.7 (-28.4 to -18.8)
	Prevalence	1666.05 (1446.59 to 1898)	1783.03 (1549.29 to 2025.26)	1551.54 (1331.27 to 1807.09)	1464.85 (1285.8 to 1670.27)	1610.47 (1398.8 to 1835.27)	1321.4 (1151.13 to 1525.66)	-12.1 (-17.6 to -5.4)	-9.7 (-17.1 to -1)	-14.8 (-22.1 to -6.2)
	Deaths	157.7 (122.6 to 184.25)	148.22 (114.06 to 181.48)	166.86 (112.84 to 208.57)	97.02 (81 to 107.65)	89.2 (73.82 to 100.15)	105.22 (81.13 to 121.4)	-38.5 (-49.8 to -24.3)	-39.8 (-52 to -22.4)	-36.9 (-52.2 to -16.9)
	DALYs	2917.74 (2326.64 to 3426.67)	2748.4 (2158.8 to 3341.48)	3074.78 (2196.27 to 3800.97)	1763.93 (1542.34 to 1951.94)	1645.05 (1414.28 to 1836.37)	1888.44 (1550.44 to 2155.6)	-39.5 (-50.9 to -26)	-40.1 (-52 to -23.2)	-38.6 (-53 to -19.4)
	YLLs	2656.63 (2070.01 to 3156.76)	2451.42 (1854.77 to 3038.8)	2848.59 (2004.95 to 3583.13)	1533.15 (1319.02 to 1706.97)	1375.09 (1175.29 to 1545.04)	1696.51 (1361.87 to 1963.32)	-42.3 (-53.9 to -27.6)	-43.9 (-56 to -26.2)	-40.4 (-55.5 to -19.8)
	YLDs	261.11 (187.29 to 338.91)	296.98 (211.06 to 382.92)	226.19 (161.63 to 294.98)	230.78 (164.78 to 297.19)	269.96 (193.34 to 347.42)	191.93 (137.78 to 248.41)	-11.6 (-17.9 to -4.5)	-9.1 (-17.6 to 0)	-15.1 (-23.7 to -5.9)
Chahar Mahaal and Bakhtiari	Incidence	155.71 (135.98 to 179.57)	154.56 (134.55 to 179.03)	157.26 (136.24 to 182.8)	136.13 (117.8 to 157.94)	138.71 (119.39 to 161.61)	133.1 (115.19 to 154.05)	-12.6 (-16.6 to -8.2)	-10.3 (-15.7 to -4.5)	-15.4 (-20.3 to -9.6)
	Prevalence	1328.66 (1166.73 to 1510.23)	1435.52 (1258.32 to 1626.76)	1224.11 (1063.26 to 1407.89)	1216.76 (1071.27 to 1394.8)	1279.96 (1121.12 to 1457.92)	1151.86 (1004.73 to 1325.21)	-8.4 (-13.9 to -3)	-10.8 (-18 to -2.3)	-5.9 (-13.9 to 2.6)
	Deaths	101.64 (80.36 to 123.57)	94.81 (68.99 to 121.46)	107.92 (81.58 to 141.27)	60.22 (50.06 to 70.25)	49.23 (38.72 to 61.99)	71.92 (57.91 to 84.63)	-40.7 (-53.6 to -23.6)	-48.1 (-62.8 to -24.3)	-33.4 (-51.2 to -9.1)
	DALYs	2015.3 (1668.67 to 2412.09)	1887.6 (1468.71 to 2375.46)	2124.09 (1645.02 to 2748.47)	1138.85 (978.2 to 1303.94)	960.81 (797.07 to 1153.69)	1326.84 (1113.43 to 1550.09)	-43.5 (-54.7 to -28.9)	-49.1 (-61.8 to -31.5)	-37.5 (-53.8 to -14.1)
	YLLs	1809.35 (1459.78 to 2218.27)	1650.23 (1244.2 to 2126.29)	1947.92 (1467.9 to 2568.48)	946.79 (800.8 to 1100.21)	745.31 (596.58 to 934.77)	1159.04 (948.6 to 1377.09)	-47.7 (-59.3 to -32.2)	-54.8 (-68.5 to -35.2)	-40.5 (-56.8 to -16)
	YLDs	205.96 (148.64 to 266.58)	237.37 (172.25 to 303.5)	176.17 (125.92 to 230.9)	192.06 (137.25 to 249.02)	215.5 (154.57 to 278.7)	167.8 (119.65 to 218.95)	-6.7 (-13.2 to -0.4)	-9.2 (-17.8 to 0.2)	-4.8 (-13 to 4.3)

Province	Measure	Age-standardized rate (per 100,000)						% Change (1990 to 2019)		
		1990			2019					
		Both	Female	Male	Both	Female	Male	Both	Female	Male
East Azarbayejan	Incidence	184.13 (161.35 to 211.76)	183.23 (160.54 to 211.69)	185.04 (161.95 to 216.42)	150.09 (130.66 to 173.16)	155.49 (135.28 to 180.05)	144.58 (125.58 to 166.08)	-18.5 (-22.1 to -14.5)	-15.1 (-20.4 to -9.1)	-21.9 (-26.5 to -16.9)
	Prevalence	1611.83 (1412.22 to 1863.53)	1778.43 (1561.14 to 2064.48)	1452.92 (1255.58 to 1692.55)	1367.02 (1204.15 to 1549.16)	1462.27 (1288.86 to 1663.34)	1271.36 (1099.42 to 1454.07)	-15.2 (-21.3 to -9.2)	-17.8 (-25.2 to -9.8)	-12.5 (-20.5 to -4.3)
	Deaths	152.91 (124.39 to 182.67)	155.56 (115.69 to 197.35)	148.21 (112.42 to 190.14)	83.22 (70.33 to 100.13)	85.75 (69.16 to 105.83)	80.64 (66.09 to 97.74)	-45.6 (-57.3 to -27.9)	-44.9 (-58.5 to -21.3)	-45.6 (-61.4 to -21.7)
	DALYs	2710.64 (2215.72 to 3230.01)	2714.7 (2130.1 to 3417.53)	2681.98 (2058.07 to 3455.66)	1447.36 (1252.38 to 1691.95)	1447.05 (1205.72 to 1737.61)	1445.89 (1210.62 to 1739.66)	-46.6 (-58.2 to -30.5)	-46.7 (-60.1 to -27.9)	-46.1 (-61.2 to -25.1)
	YLLs	2457.51 (1990.03 to 2983.47)	2416.89 (1821.07 to 3143.96)	2470.71 (1860.76 to 3259.41)	1231.96 (1045.33 to 1480.63)	1201.13 (974.59 to 1487)	1261.27 (1035.3 to 1548.03)	-49.9 (-61.9 to -32.5)	-50.3 (-64.1 to -29.6)	-49 (-63.7 to -25.7)
	YLDs	253.13 (183.19 to 328.97)	297.81 (214.02 to 383.92)	211.26 (149.77 to 280.07)	215.4 (153.83 to 276.65)	245.93 (172.94 to 319.33)	184.62 (131.09 to 238.51)	-14.9 (-21.5 to -8.3)	-17.4 (-25.4 to -8.7)	-12.6 (-21.3 to -3.1)
Fars	Incidence	163.87 (143.18 to 188.99)	166.53 (144.2 to 192.13)	162.11 (141.32 to 187.67)	142.37 (123.78 to 164.69)	147.86 (127.37 to 171.59)	137.13 (118.89 to 158.69)	-13.1 (-16.7 to -9.3)	-11.2 (-17 to -4.8)	-15.4 (-20.7 to -10)
	Prevalence	1438.64 (1257.54 to 1650.3)	1614.36 (1408.71 to 1848.8)	1262.87 (1095.23 to 1467.53)	1294.3 (1139.04 to 1463.19)	1392.18 (1215.33 to 1578.8)	1198.95 (1042.76 to 1366.46)	-10 (-15.9 to -3.2)	-13.8 (-21.4 to -4.9)	-5.1 (-14.9 to 4.9)
	Deaths	122.67 (98.29 to 145.21)	121.1 (93.95 to 152.67)	122.15 (92.18 to 153.8)	82.67 (69.07 to 94.92)	83.8 (67.31 to 99.34)	82.19 (64.81 to 99.82)	-32.6 (-46.3 to -16)	-30.8 (-47.3 to -8.7)	-32.7 (-50.6 to -8)
	DALYs	2251.2 (1871.66 to 2708.08)	2197.31 (1728.19 to 2775)	2278 (1758.54 to 2838.25)	1478.33 (1280.65 to 1680.05)	1434.27 (1204.76 to 1663.82)	1526.25 (1251.57 to 1844.96)	-34.3 (-48.3 to -18.4)	-34.7 (-49.7 to -14.2)	-33 (-51.2 to -7.3)
	YLLs	2026.66 (1657.28 to 2479.57)	1930.01 (1453.03 to 2461.54)	2096.32 (1588.46 to 2676.88)	1274.84 (1088.16 to 1466.36)	1201.08 (983.08 to 1427.41)	1351.99 (1083.03 to 1666.46)	-37.1 (-51.7 to -19.4)	-37.8 (-54.4 to -14.1)	-35.5 (-54.4 to -8.1)
	YLDs	224.53 (160.62 to 290.57)	267.29 (190.98 to 349.55)	181.68 (129.44 to 235.52)	203.49 (146.24 to 262.62)	233.19 (167.89 to 304.75)	174.27 (124.53 to 228.53)	-9.4 (-15.7 to -2.3)	-12.8 (-20.6 to -3.5)	-4.1 (-14 to 6.6)

Province	Measure	Age-standardized rate (per 100,000)						% Change (1990 to 2019)		
		1990			2019					
		Both	Female	Male	Both	Female	Male	Both	Female	Male
Gilan	Incidence	180.64 (156.95 to 207.93)	183.09 (158.83 to 211.93)	179.8 (155.83 to 207.32)	155.98 (136.43 to 179.11)	162.32 (140.71 to 188.33)	149.68 (129.72 to 172.73)	-13.7 (-17.3 to -9.9)	-11.3 (-17.1 to -5.1)	-16.8 (-21.4 to -12.4)
	Prevalence	1628.01 (1433.39 to 1850.91)	1811.22 (1581.49 to 2060.53)	1426.27 (1239.35 to 1652.07)	1417.78 (1251.59 to 1608.08)	1535.84 (1348.44 to 1748.47)	1298.58 (1140.5 to 1493.06)	-12.9 (-18.1 to -7.4)	-15.2 (-22.1 to -7.9)	-9 (-16.9 to -0.6)
	Deaths	150.01 (117.83 to 177.28)	152.72 (118.8 to 188.39)	142.05 (101.87 to 182.33)	82.42 (70.74 to 94)	88.68 (72.54 to 104.01)	76.85 (62.39 to 91.98)	-45.1 (-55.7 to -30)	-41.9 (-56.8 to -21.8)	-45.9 (-59.7 to -25.8)
	DALYs	2832.16 (2294.73 to 3347.33)	2852.51 (2289.04 to 3494.72)	2756.47 (2064.81 to 3509.2)	1554.69 (1366.94 to 1749.82)	1586.18 (1330.52 to 1838.63)	1526.75 (1275.92 to 1814.2)	-45.1 (-55.1 to -31.8)	-44.4 (-58.2 to -26.9)	-44.6 (-58.5 to -25.3)
	YLLs	2576.26 (2037.66 to 3098.97)	2551.34 (1964.58 to 3200.44)	2550.81 (1877.02 to 3313.69)	1332.2 (1155.73 to 1517.88)	1329.21 (1097.95 to 1570.59)	1339.24 (1090.72 to 1618.02)	-48.3 (-58.7 to -33.8)	-47.9 (-62 to -28.7)	-47.5 (-61.7 to -27.3)
	YLDs	255.9 (182.58 to 331.36)	301.17 (215.81 to 389.87)	205.66 (147.58 to 267.09)	222.49 (158.98 to 286.45)	256.97 (184.71 to 331.62)	187.5 (134.55 to 243.35)	-13.1 (-18.8 to -7)	-14.7 (-22.2 to -6)	-8.8 (-17.8 to -0.2)
Golestan	Incidence	189.74 (164.22 to 219.79)	192.95 (166.94 to 222.91)	186.96 (162.19 to 217.63)	171.54 (148.87 to 201.32)	179.87 (154.77 to 212.55)	162.87 (140.52 to 190.92)	-9.6 (-13.3 to -5.5)	-6.8 (-12.3 to -1.1)	-12.9 (-18.2 to -7)
	Prevalence	1687.3 (1483.38 to 1911.74)	1890.45 (1652.46 to 2135.85)	1480.07 (1290.47 to 1714.45)	1574.59 (1377.1 to 1811.41)	1717.74 (1501.43 to 1990.73)	1425.42 (1228.25 to 1657.96)	-6.7 (-13.5 to -0.4)	-9.1 (-18.1 to -0.5)	-3.7 (-12.6 to 6.2)
	Deaths	145.13 (114.72 to 170.98)	141.37 (108.3 to 170.84)	146.82 (109.76 to 187.34)	89.49 (77.83 to 99.04)	84.49 (71.12 to 98.18)	95.08 (79.46 to 111.01)	-38.3 (-49.4 to -21)	-40.2 (-54.4 to -18)	-35.2 (-52.2 to -11.4)
	DALYs	2962.58 (2395.24 to 3503.7)	2903.95 (2295.61 to 3509.3)	2988.28 (2275.79 to 3814.03)	1808.62 (1605.2 to 1993.81)	1713.23 (1471.97 to 1965.1)	1911.68 (1632.63 to 2222.64)	-39 (-49.3 to -23.2)	-41 (-54.8 to -21.7)	-36 (-52.2 to -13.4)
	YLLs	2699.47 (2136.54 to 3229.2)	2590.67 (1968.42 to 3211.88)	2775.6 (2080.26 to 3610.3)	1561.5 (1365.08 to 1735.11)	1426.56 (1203.35 to 1663.08)	1706.04 (1436.52 to 2007.08)	-42.2 (-52.7 to -25.2)	-44.9 (-59.3 to -23.7)	-38.5 (-54.9 to -14.3)
	YLDs	263.11 (188.06 to 341.64)	313.28 (222.33 to 405.74)	212.68 (150.58 to 280.31)	247.12 (176.77 to 322.27)	286.67 (204.94 to 377.13)	205.64 (143.97 to 266.14)	-6.1 (-13.7 to 1.2)	-8.5 (-18.1 to 0.7)	-3.3 (-12.5 to 7.4)

Province	Measure	Age-standardized rate (per 100,000)						% Change (1990 to 2019)		
		1990			2019					
		Both	Female	Male	Both	Female	Male	Both	Female	Male
Hamadan	Incidence	169.42 (148.65 to 193.72)	173.34 (150.89 to 200.53)	165.58 (144.14 to 191)	149.31 (129.43 to 171.35)	156.18 (134.24 to 180.86)	142.78 (123.14 to 165.43)	-11.9 (-15.8 to -7.5)	-9.9 (-15.8 to -2.9)	-13.8 (-19.1 to -8.2)
	Prevalence	1466.56 (1288.8 to 1680.4)	1661.26 (1454.21 to 1904.15)	1279.48 (1117.68 to 1476.35)	1322.57 (1150.36 to 1512.61)	1451.03 (1260 to 1668.18)	1195.15 (1037.23 to 1380.08)	-9.8 (-15.6 to -3.3)	-12.7 (-20.2 to -4.1)	-6.6 (-14.7 to 2.7)
	Deaths	135.63 (104.09 to 160.56)	142.71 (111.07 to 178.82)	126.28 (85.76 to 160.69)	73.08 (60.74 to 82.59)	77.36 (63.97 to 91.11)	70.85 (55.12 to 84.72)	-46.1 (-57.3 to -33.5)	-45.8 (-59 to -26.5)	-43.9 (-59.2 to -23.6)
	DALYs	2718.38 (2187.33 to 3218.68)	2746.34 (2204.62 to 3412.19)	2663.2 (1949.3 to 3348.62)	1463.31 (1266.4 to 1641.54)	1443.58 (1234.7 to 1677.51)	1497.53 (1216.94 to 1782.78)	-46.2 (-56.9 to -34.2)	-47.4 (-59.5 to -32)	-43.8 (-58.4 to -25)
	YLLs	2490.52 (1966.91 to 2981.08)	2471.86 (1936.93 to 3144.65)	2479.61 (1762.25 to 3178.27)	1254.86 (1059.85 to 1426.27)	1199.81 (1000.43 to 1418.64)	1324.51 (1046.09 to 1595.87)	-49.6 (-60.4 to -36.8)	-51.5 (-64.1 to -34.4)	-46.6 (-61.6 to -26.6)
	YLDs	227.87 (162.45 to 293.84)	274.47 (194.64 to 353.63)	183.59 (131.02 to 238.28)	208.45 (149.09 to 269.49)	243.77 (174.06 to 312.53)	173.02 (123.43 to 224.22)	-8.5 (-14.8 to -1.1)	-11.2 (-19.1 to -2.2)	-5.8 (-14.8 to 4.1)
Hormozgan	Incidence	173.27 (151.22 to 198.94)	170.59 (148.38 to 195.29)	176.43 (153.02 to 204.64)	143.1 (124.54 to 165.11)	147.11 (127.31 to 169.57)	139.42 (120.62 to 162.11)	-17.4 (-20.8 to -13.5)	-13.8 (-18.7 to -8.3)	-21 (-25.5 to -15.4)
	Prevalence	1470.83 (1292.25 to 1666.88)	1606.24 (1406.54 to 1811.07)	1343.3 (1168.39 to 1546.87)	1294.84 (1141.54 to 1469.71)	1376.98 (1201.76 to 1558.95)	1216.4 (1051.65 to 1395.19)	-12 (-17.5 to -6.5)	-14.3 (-20.8 to -7.1)	-9.4 (-17.5 to -1)
	Deaths	134.65 (104.3 to 162.46)	120.78 (91.36 to 147.94)	148.74 (104.99 to 191.7)	66.72 (56.68 to 75.76)	67.92 (55.99 to 77.7)	67.25 (54.29 to 80.76)	-50.4 (-60.9 to -35.3)	-43.8 (-55.4 to -23.6)	-54.8 (-67.9 to -35.7)
	DALYs	2689.9 (2041.09 to 3271.32)	2450.49 (1854.13 to 3006.77)	2901.93 (2055.41 to 3744.73)	1348.39 (1190.43 to 1509.59)	1295.47 (1108.31 to 1480.64)	1417.17 (1186.07 to 1683.22)	-49.9 (-60.2 to -32.5)	-47.1 (-58.4 to -28.6)	-51.2 (-64.5 to -29)
	YLLs	2462.04 (1814.51 to 3025.85)	2185.5 (1595.11 to 2725.31)	2708.46 (1865.02 to 3584.95)	1145.63 (994.05 to 1306.84)	1064.95 (891.43 to 1227.53)	1241.72 (1013.42 to 1497.87)	-53.5 (-63.7 to -35.4)	-51.3 (-63.2 to -30.3)	-54.2 (-67.7 to -30.9)
	YLDs	227.86 (161.87 to 293.63)	264.98 (188.76 to 342.47)	193.46 (136.52 to 252.08)	202.75 (145.36 to 262.23)	230.53 (167.36 to 297.36)	175.46 (126.03 to 227.9)	-11 (-16.7 to -4.8)	-13 (-20.5 to -4.8)	-9.3 (-17.7 to 0.3)

Province	Measure	Age-standardized rate (per 100,000)						% Change (1990 to 2019)		
		1990			2019					
		Both	Female	Male	Both	Female	Male	Both	Female	Male
Ilam	Incidence	142.31 (125.52 to 163.34)	137.32 (120.69 to 157.82)	146.26 (128.7 to 169.64)	130.73 (114.36 to 150.64)	137.91 (120.49 to 158.72)	124.36 (107.63 to 144.07)	-8.1 (-12.9 to -3.5)	0.4 (-6.2 to 8)	-15 (-20.2 to -9.5)
	Prevalence	1156.24 (1018.66 to 1312.89)	1221.7 (1073.27 to 1390.62)	1099.16 (956.73 to 1272.42)	1138.69 (1000.27 to 1304.64)	1239.96 (1084.22 to 1417.29)	1042.98 (908.07 to 1199.19)	-1.5 (-7.5 to 4.9)	1.5 (-6.9 to 11.2)	-5.1 (-13.4 to 3)
	Deaths	89.32 (72.11 to 106.59)	78.36 (57.58 to 97.2)	98.99 (77.5 to 126.08)	63.73 (55.54 to 71.03)	59.31 (49.31 to 68.81)	66.9 (57.31 to 77.53)	-28.6 (-41.9 to -9.2)	-24.3 (-41.7 to 7.8)	-32.4 (-48.3 to -9.6)
	DALYs	1764.74 (1450.48 to 2118.4)	1620.34 (1236.1 to 2003.56)	1881.11 (1494.39 to 2373.08)	1194.66 (1065.64 to 1317.77)	1141.11 (982.28 to 1296.77)	1235.51 (1061.55 to 1427.31)	-32.3 (-44.2 to -15.3)	-29.6 (-46.4 to -4.1)	-34.3 (-50.3 to -13.2)
	YLLs	1586.09 (1274.09 to 1917.83)	1418.76 (1053.69 to 1794.25)	1721.12 (1333.85 to 2209.36)	1015.11 (907.19 to 1125.4)	932.97 (788.07 to 1082.58)	1083.3 (923.24 to 1266.01)	-36 (-48.3 to -17.4)	-34.2 (-50.7 to -6)	-37.1 (-53.4 to -13.6)
	YLDs	178.65 (128.58 to 231.1)	201.58 (144.88 to 259.9)	159.99 (114.98 to 208.46)	179.55 (129.79 to 233.37)	208.14 (151.56 to 270.41)	152.21 (109.51 to 197.95)	0.5 (-6.3 to 7.5)	3.3 (-5.6 to 13.8)	-4.9 (-13.8 to 4.3)
Isfahan	Incidence	148.18 (130.23 to 170.21)	145.43 (126.83 to 168.48)	151.92 (133.55 to 174.56)	117.02 (102.15 to 134.41)	120.14 (103.93 to 138.38)	114.32 (99.54 to 132.29)	-21 (-24.7 to -17.4)	-17.4 (-22.6 to -12.2)	-24.7 (-29.7 to -19.9)
	Prevalence	1344.14 (1169.41 to 1535.64)	1473.1 (1274.36 to 1688.05)	1210.34 (1051.31 to 1415.14)	1110.13 (976 to 1264.84)	1196.29 (1053.45 to 1369.95)	1028.41 (891.09 to 1183.78)	-17.4 (-22.8 to -11.4)	-18.8 (-25.3 to -10.8)	-15 (-22 to -7.7)
	Deaths	107.9 (84.25 to 133.46)	112.98 (85.63 to 145.94)	98.93 (68.96 to 130.01)	61.84 (52.24 to 71.3)	67.59 (54.34 to 80.72)	57.42 (46.09 to 68.61)	-42.7 (-55 to -25.3)	-40.2 (-56.3 to -17.6)	-42 (-59.6 to -17.4)
	DALYs	1947.63 (1538.61 to 2388.68)	1979.88 (1510.11 to 2532.89)	1872.38 (1365.5 to 2413.42)	1113.24 (966.44 to 1266.06)	1140.58 (964.82 to 1341.19)	1095.79 (904.58 to 1299.88)	-42.8 (-55.2 to -26.1)	-42.4 (-57.4 to -21.8)	-41.5 (-58 to -17.8)
	YLLs	1736.25 (1321.61 to 2169.14)	1734.24 (1270.6 to 2289.96)	1696.56 (1185.29 to 2225.07)	938.3 (800.54 to 1087.36)	939.23 (764.78 to 1141.15)	946.16 (762.76 to 1141.17)	-46 (-58.7 to -27.1)	-45.8 (-61.6 to -22.9)	-44.2 (-61.8 to -18.3)
	YLDs	211.38 (150.42 to 273.61)	245.64 (174.91 to 318.21)	175.82 (125.2 to 231.69)	174.94 (125.25 to 222.68)	201.35 (143.67 to 257.08)	149.62 (106.73 to 194.38)	-17.2 (-22.9 to -10.4)	-18 (-25.4 to -9.5)	-14.9 (-22.3 to -6.8)

Province	Measure	Age-standardized rate (per 100,000)						% Change (1990 to 2019)		
		1990			2019					
		Both	Female	Male	Both	Female	Male	Both	Female	Male
Kerman	Incidence	147.48 (127.03 to 171.4)	148.69 (127.61 to 174.04)	147.01 (126.12 to 171.62)	121.6 (103.6 to 144.65)	127.25 (108.6 to 150.04)	116.15 (98.61 to 138.4)	-17.5 (-21.5 to -13.1)	-14.4 (-19.9 to -8.1)	-21 (-26.8 to -14.8)
	Prevalence	1344.3 (1169.85 to 1528.2)	1499.01 (1302.69 to 1700.23)	1191.66 (1026.91 to 1369.12)	1098.24 (958.23 to 1248.82)	1201.23 (1041.7 to 1383.34)	998.27 (859.55 to 1142.17)	-18.3 (-23.3 to -12.5)	-19.9 (-26.7 to -12.5)	-16.2 (-23.2 to -8.1)
	Deaths	120.55 (98.56 to 142.78)	117.96 (92 to 146.37)	121.57 (96.05 to 155.37)	64.8 (55.8 to 75.1)	67.21 (55.64 to 79.12)	62.65 (51.92 to 76.36)	-46.3 (-56.3 to -32.8)	-43 (-56.9 to -23.9)	-48.5 (-62 to -31)
	DALYs	2313.38 (1935.74 to 2774.54)	2232.87 (1781.82 to 2798.07)	2368.44 (1902.41 to 3015.48)	1194.79 (1054.87 to 1371.38)	1191.8 (1020.91 to 1382.43)	1199.57 (1020.25 to 1439.33)	-48.4 (-57.6 to -36.8)	-46.6 (-59 to -31.3)	-49.4 (-62.7 to -32.3)
	YLLs	2104.88 (1720.71 to 2562.01)	1985.83 (1527.46 to 2544.92)	2197.68 (1723.05 to 2830.88)	1023.73 (888.67 to 1195.38)	992.62 (836.05 to 1170.34)	1055.98 (876.95 to 1297.12)	-51.4 (-61 to -39)	-50 (-63.3 to -33.3)	-52 (-65.4 to -34.3)
	YLDs	208.5 (151.12 to 267.46)	247.04 (180.14 to 319.09)	170.77 (122.47 to 220.75)	171.07 (123.02 to 220.14)	199.18 (142.66 to 257.68)	143.59 (103.08 to 185.36)	-18 (-23.5 to -11.9)	-19.4 (-26.9 to -11.4)	-15.9 (-23.6 to -7.6)
Kermanshah	Incidence	182.84 (160.03 to 209.44)	181.58 (159.36 to 207.81)	183.4 (159.97 to 213.44)	152.56 (132.47 to 175.93)	159.66 (138.22 to 184.53)	145.84 (126.02 to 170.37)	-16.6 (-20 to -13.2)	-12.1 (-17.7 to -6)	-20.5 (-25.2 to -15)
	Prevalence	1543.83 (1360.85 to 1771.84)	1711.77 (1507.91 to 1961.35)	1394.08 (1210.24 to 1608.61)	1375.96 (1203.72 to 1557.89)	1480.92 (1294.67 to 1685)	1271.86 (1100.42 to 1446.46)	-10.9 (-16.5 to -4.6)	-13.5 (-20.3 to -6.1)	-8.8 (-17.2 to 0.4)
	Deaths	154.79 (123.68 to 184.58)	146.33 (112.35 to 180.97)	160.6 (122.71 to 201.34)	80.97 (69.09 to 93.24)	86.16 (70.93 to 101.89)	77.04 (61.58 to 92.17)	-47.7 (-58.3 to -33.7)	-41.1 (-55.7 to -19.1)	-52 (-64.2 to -34.9)
	DALYs	3086.14 (2536.2 to 3693.85)	2882.54 (2275.39 to 3551.84)	3236.46 (2513.24 to 4093.43)	1566.05 (1367.44 to 1784.27)	1598.13 (1353.43 to 1876.4)	1543.62 (1283.15 to 1824.02)	-49.3 (-59.4 to -36.1)	-44.6 (-58.2 to -25.8)	-52.3 (-63.9 to -35.9)
	YLLs	2846.82 (2291.43 to 3447.6)	2599.02 (1954.25 to 3241.58)	3035.54 (2325.5 to 3872.99)	1350.1 (1162.52 to 1549.74)	1350.61 (1113.91 to 1606.89)	1359.39 (1105.5 to 1635.53)	-52.6 (-62.7 to -39)	-48 (-62.4 to -27.9)	-55.2 (-67.1 to -37.9)
	YLDs	239.32 (171.59 to 309.07)	283.52 (201.72 to 361.99)	200.92 (143.48 to 259.92)	215.95 (153.94 to 279.07)	247.53 (177.47 to 321.81)	184.23 (130.72 to 239.37)	-9.8 (-15.9 to -3.1)	-12.7 (-20.2 to -4.9)	-8.3 (-17.3 to 1.4)

Province	Measure	Age-standardized rate (per 100,000)						% Change (1990 to 2019)		
		1990			2019					
		Both	Female	Male	Both	Female	Male	Both	Female	Male
Khorasan -e Razavi	Incidence	184.85 (163.19 to 210.94)	183.16 (160.4 to 209.23)	186.84 (164.72 to 213.65)	138.43 (121.58 to 158.84)	142.06 (124.57 to 163.66)	134.81 (117.19 to 156.53)	-25.1 (-28.4 to -22)	-22.4 (-27.1 to -16.9)	-27.8 (-32.1 to -23.5)
	Prevalence	1495.06 (1314.82 to 1711.99)	1650.86 (1439.81 to 1881.25)	1345.32 (1170.78 to 1562.17)	1262.1 (1110.36 to 1440)	1339.56 (1177.42 to 1514.88)	1183.91 (1026.11 to 1369.32)	-15.6 (-20.7 to -10.5)	-18.9 (-25.3 to -12.2)	-12 (-19 to - 4.4)
	Deaths	153.25 (127.23 to 181.62)	147.38 (114.27 to 183.21)	158.18 (122.97 to 201.41)	78.55 (67.85 to 90.38)	79.16 (64.96 to 93.87)	78.05 (65.67 to 93.01)	-48.7 (-59 to -36.1)	-46.3 (-60.1 to -28)	-50.7 (-63.5 to -33.4)
	DALYs	2938.15 (2465.46 to 3514.74)	2832.85 (2259.98 to 3534.98)	3023.07 (2381.61 to 3840.67)	1464.8 (1284.94 to 1659.98)	1441.42 (1213.98 to 1697.58)	1489.87 (1277.44 to 1756.61)	-50.1 (-60.4 to -39)	-49.1 (-62.2 to -33.6)	-50.7 (-63.3 to -34.4)
	YLLs	2707.48 (2233.28 to 3265.8)	2562.17 (1982.18 to 3257.41)	2830.37 (2189.09 to 3660.02)	1269.19 (1099.45 to 1457.06)	1220.12 (998.93 to 1450.9)	1320.5 (1099.66 to 1590.95)	-53.1 (-63.6 to -41.3)	-52.4 (-66.3 to -35.6)	-53.3 (-65.9 to -36.5)
	YLDs	230.67 (165.43 to 296.8)	270.69 (194.19 to 347.37)	192.7 (137.67 to 254.05)	195.61 (141.17 to 250.6)	221.3 (158.96 to 286.28)	169.38 (120.34 to 217.57)	-15.2 (-20.7 to -9.5)	-18.2 (-25.2 to -11.3)	-12.1 (-19.6 to -3.4)
Khuzestan	Incidence	173.4 (151.04 to 201.94)	174.33 (151.39 to 201.56)	173.45 (149.01 to 205.05)	160.45 (138.97 to 186.11)	166.94 (143.35 to 195.76)	153.95 (133.14 to 178.63)	-7.5 (-11.4 to -2.6)	-4.2 (-9.8 to 2.7)	-11.2 (-16.2 to -4.6)
	Prevalence	1564.57 (1375.03 to 1801.86)	1721.16 (1516.28 to 1965.69)	1408.1 (1215.06 to 1660.98)	1490.81 (1301.9 to 1695.43)	1609.54 (1393.16 to 1838.46)	1372.42 (1188.97 to 1575.17)	-4.7 (-11.7 to 2.8)	-6.5 (-15.4 to 3)	-2.5 (-11.3 to 8.6)
	Deaths	132.59 (108.67 to 156.62)	128.56 (101.52 to 158.64)	135.47 (102.98 to 169.97)	85.34 (72.88 to 97.51)	88.38 (72.95 to 104.24)	82.76 (66.09 to 99.29)	-35.6 (-48.1 to -18.1)	-31.3 (-48.1 to -8.5)	-38.9 (-55.1 to -17.4)
	DALYs	2540.74 (2129.69 to 2989.78)	2468.92 (2005.8 to 3025.54)	2592.03 (2014.22 to 3230.48)	1594.84 (1401.27 to 1802.46)	1612.11 (1356.05 to 1875.95)	1581.1 (1306.31 to 1868)	-37.2 (-49.4 to -22.3)	-34.7 (-50.1 to -15.8)	-39 (-54.8 to - 18.7)
	YLLs	2298.52 (1882.8 to 2747.74)	2185.75 (1726.73 to 2768.62)	2390.77 (1822.33 to 3029.39)	1361.65 (1180.05 to 1554.98)	1343.56 (1102.52 to 1602.59)	1383.44 (1112.05 to 1673.59)	-40.8 (-53.1 to -24.7)	-38.5 (-55 to -16.7)	-42.1 (-58.6 to -20.1)
	YLDs	242.22 (173.04 to 313.72)	283.17 (202.23 to 362.88)	201.26 (143.72 to 265.2)	233.19 (168.3 to 302.63)	268.55 (193.19 to 348.79)	197.65 (139.3 to 258.26)	-3.7 (-11.2 to 4.1)	-5.2 (-14 to 4.2)	-1.8 (-11.4 to 9.4)

Province	Measure	Age-standardized rate (per 100,000)						% Change (1990 to 2019)		
		1990			2019					
		**Both**	**Female**	**Male**	**Both**	**Female**	**Male**	**Both**	**Female**	**Male**
**Kohgiluyeh and Boyer- Ahmad**	**Incidence**	161.15 (140.96 to 185.11)	157.63 (137.35 to 181.4)	165.5 (144.22 to 191.3)	140.41 (122.21 to 161.61)	148.88 (128.94 to 172.32)	133.58 (116.14 to 153.36)	-12.9 (-16.9 to -8.8)	-5.5 (-11.6 to 0.7)	-19.3 (-24.3 to -14)
	**Prevalence**	1363.52 (1197.36 to 1556.72)	1463.38 (1286.07 to 1659.87)	1269.18 (1102.81 to 1459.3)	1262 (1114.76 to 1428.2)	1382.07 (1211.93 to 1562.01)	1156.98 (1008.71 to 1322.76)	-7.4 (-12.8 to -0.7)	-5.6 (-12.9 to 3.2)	-8.8 (-16.2 to -0.2)
	**Deaths**	115.06 (89.26 to 139.44)	122.99 (92.34 to 157.24)	104.1 (76.45 to 135.86)	66.93 (53.79 to 78.74)	70.05 (55.04 to 86.12)	65.03 (49.24 to 82.04)	-41.8 (-55.9 to -24.3)	-43 (-59.2 to -20.4)	-37.5 (-56.2 to -10.4)
	**DALYs**	2263.46 (1819.24 to 2731.54)	2352.74 (1849.75 to 3023.93)	2137.29 (1612.52 to 2765.84)	1296.61 (1099.12 to 1508.21)	1325.79 (1087.16 to 1582.01)	1282.14 (996.51 to 1599.14)	-42.7 (-56 to -27.5)	-43.6 (-58.5 to -24.3)	-40 (-57.1 to -16.8)
	**YLLs**	2053.19 (1617.55 to 2514.96)	2112.02 (1609.96 to 2752.53)	1955.75 (1438.71 to 2574.03)	1098.36 (899.58 to 1295)	1093.49 (857.93 to 1354.65)	1113.99 (829.62 to 1428.32)	-46.5 (-60.3 to -30.6)	-48.2 (-63.2 to -27.6)	-43 (-61.2 to -18.4)
	**YLDs**	210.27 (148.75 to 269.29)	240.72 (168.63 to 308.28)	181.54 (128.74 to 237.64)	198.25 (142.49 to 255.81)	232.31 (167.16 to 300.54)	168.16 (118.62 to 219.86)	-5.7 (-11.6 to 1.4)	-3.5 (-11.8 to 6.2)	-7.4 (-16.1 to 2.5)
**Kurdistan**	**Incidence**	165.01 (144.74 to 189.27)	163.82 (143.97 to 188.85)	165.62 (144.61 to 191.34)	138.09 (120.64 to 159.31)	141.98 (123.61 to 163.13)	134.74 (117.67 to 156.22)	-16.3 (-19.9 to -12.3)	-13.3 (-18.4 to -7.2)	-18.6 (-23.6 to -13.5)
	**Prevalence**	1373.53 (1211.71 to 1559.64)	1505.28 (1324.5 to 1703.74)	1254.98 (1089.1 to 1435.41)	1208.36 (1072.41 to 1373.41)	1294.73 (1134.84 to 1478.8)	1125.66 (992.78 to 1292.43)	-12 (-17.3 to -6.5)	-14 (-21.4 to -5.9)	-10.3 (-17.5 to -1.4)
	**Deaths**	134.66 (109.33 to 158.85)	124.57 (96.54 to 155.4)	143.91 (110.04 to 178.78)	63.82 (54.84 to 72.43)	65.65 (54.46 to 75.44)	63.02 (51.15 to 75.78)	-52.6 (-62 to -40.6)	-47.3 (-59.6 to -30.4)	-56.2 (-67.9 to -41.1)
	**DALYs**	2690.43 (2258.61 to 3154.73)	2548.14 (2066.61 to 3127.48)	2807.41 (2217.01 to 3469.48)	1252.26 (1106.9 to 1413.87)	1230.85 (1045.19 to 1412.51)	1283.22 (1066.02 to 1523.62)	-53.5 (-62.1 to -43.4)	-51.7 (-63.3 to -38.2)	-54.3 (-66.1 to -38.6)
	**YLLs**	2477.45 (2044.18 to 2950.05)	2298.58 (1826.15 to 2896.28)	2626.17 (2025.91 to 3297.5)	1062.47 (924.74 to 1204.89)	1013.74 (848.26 to 1173.27)	1120.04 (911.59 to 1359.09)	-57.1 (-66.2 to -46.5)	-55.9 (-67.2 to -41.9)	-57.4 (-69.1 to -40.8)
	**YLDs**	212.98 (154.08 to 275.68)	249.55 (176.04 to 320.34)	181.24 (128.11 to 236.02)	189.79 (136.08 to 244.44)	217.11 (155.47 to 282.23)	163.18 (116.97 to 210.53)	-10.9 (-17.1 to -4.7)	-13 (-21.4 to -4)	-10 (-17.9 to -0.2)

Province	Measure	Age-standardized rate (per 100,000)						% Change (1990 to 2019)		
		1990			2019					
		Both	Female	Male	Both	Female	Male	Both	Female	Male
Lorestan	Incidence	173.16 (152.2 to 198.72)	169.64 (148.42 to 195.49)	176.21 (154.13 to 203.91)	145.96 (126.92 to 168.65)	147.29 (127.96 to 170.75)	144.77 (125.56 to 168.02)	-15.7 (-19.2 to -12.1)	-13.2 (-18.5 to -8.1)	-17.8 (-22.5 to -12.7)
	Prevalence	1457.01 (1280.62 to 1661.23)	1573.62 (1382.51 to 1792.86)	1347.81 (1178 to 1555.99)	1291.05 (1136.74 to 1469.85)	1349.08 (1183.11 to 1536.77)	1233.59 (1069.24 to 1420)	-11.4 (-16.6 to -6.1)	-14.3 (-20.6 to -7.3)	-8.5 (-16.4 to -0.2)
	Deaths	141.78 (112.84 to 168.41)	128.81 (96.01 to 157.66)	153.14 (112.2 to 194.54)	82.52 (65.33 to 95.51)	68.15 (51.58 to 80.49)	97.26 (71.5 to 116.05)	-41.8 (-53.5 to -26.5)	-47.1 (-61.9 to -27.6)	-36.5 (-54.3 to -13.4)
	DALYs	2769.01 (2266.47 to 3315.87)	2518.29 (1962.25 to 3096.35)	2975.44 (2285.47 to 3773.22)	1555.8 (1283.96 to 1781.59)	1294.98 (1026.6 to 1504.23)	1829.3 (1441.16 to 2175.88)	-43.8 (-54.6 to -29.9)	-48.6 (-62.5 to -31.8)	-38.5 (-54 to -16.3)
	YLLs	2543.77 (2036.77 to 3091.61)	2257.67 (1682.57 to 2838.86)	2782.2 (2085.51 to 3564.2)	1352.87 (1104.77 to 1568.51)	1068.34 (813.14 to 1268.9)	1650.57 (1253.82 to 1992.49)	-46.8 (-58.3 to -31.9)	-52.7 (-67.2 to -34.3)	-40.7 (-56.7 to -16.7)
	YLDs	225.24 (161.17 to 290.43)	260.62 (187.79 to 339.19)	193.24 (138.92 to 252.22)	202.93 (147.51 to 263.05)	226.65 (163.42 to 294.15)	178.72 (129.05 to 233.13)	-9.9 (-16.1 to -3.6)	-13 (-20.2 to -5.2)	-7.5 (-16.6 to 1.7)
Markazi	Incidence	175.83 (153.95 to 203.45)	175.72 (153.72 to 202.47)	175.74 (152.98 to 204.62)	139.36 (121.77 to 160.87)	145.1 (125.99 to 168.23)	133.52 (116.42 to 155.17)	-20.7 (-24.2 to -17.2)	-17.4 (-22.1 to -12.6)	-24 (-28.8 to -19)
	Prevalence	1521.93 (1330.68 to 1715.19)	1677.94 (1463.29 to 1874.75)	1364.39 (1185.93 to 1572.52)	1239.96 (1084.17 to 1410.56)	1342.11 (1176.79 to 1524.25)	1136.72 (985.76 to 1299.48)	-18.5 (-23.6 to -13.1)	-20 (-26.7 to -13.4)	-16.7 (-23.4 to -8.9)
	Deaths	138.24 (114.23 to 164.36)	138.32 (106.03 to 174.36)	136.33 (103.71 to 172.53)	61.37 (53.11 to 70.36)	63.92 (52.76 to 76.55)	59.33 (47.86 to 72.07)	-55.6 (-64.9 to -43.9)	-53.8 (-65.3 to -36.5)	-56.5 (-67.9 to -40.4)
	DALYs	2634.96 (2231.32 to 3120.6)	2572.52 (2057.34 to 3176.18)	2675.11 (2098.38 to 3356.93)	1192.33 (1052.03 to 1356.25)	1188.7 (1011.48 to 1390.92)	1197.97 (994.9 to 1433.89)	-54.7 (-64 to -44)	-53.8 (-64.9 to -38.8)	-55.2 (-66.4 to -39.2)
	YLLs	2399.15 (2000.77 to 2874.87)	2295.61 (1794.49 to 2909.78)	2480.18 (1902.37 to 3175.78)	998.23 (868.74 to 1155.99)	964.72 (801.97 to 1160.81)	1034.27 (835.21 to 1271.34)	-58.4 (-67.8 to -47.4)	-58 (-69 to -41.9)	-58.3 (-69.7 to -41.5)
	YLDs	235.81 (168.68 to 305.62)	276.91 (196.14 to 356.22)	194.94 (138.92 to 253.85)	194.1 (138.14 to 249.1)	223.97 (159.04 to 287.18)	163.7 (115.74 to 210.86)	-17.7 (-23.4 to -11.5)	-19.1 (-26.4 to -11.6)	-16 (-23.7 to -6.7)

Province	Measure	Age-standardized rate (per 100,000)						% Change (1990 to 2019)		
		1990			2019					
		Both	Female	Male	Both	Female	Male	Both	Female	Male
Mazandaran	Incidence	177.05 (154.58 to 203.56)	180.74 (157.46 to 207.67)	174.64 (152.85 to 202.01)	149.37 (131.38 to 172.32)	156.82 (137.02 to 181.48)	141.71 (123.36 to 163.57)	-15.6 (-18.7 to -11.7)	-13.2 (-18.6 to -7.4)	-18.9 (-23.2 to -14)
	Prevalence	1577.69 (1383.71 to 1793.68)	1752.99 (1525.44 to 1997.11)	1398.76 (1210.73 to 1611.33)	1347.39 (1195.3 to 1528.34)	1465.54 (1293.75 to 1671.9)	1226.87 (1081.27 to 1396.58)	-14.6 (-19.7 to -8.7)	-16.4 (-23.1 to -8.9)	-12.3 (-19.2 to -3.5)
	Deaths	117.39 (94.52 to 139.6)	123.32 (97.37 to 154.11)	108.73 (80.2 to 139.24)	64.21 (55.28 to 74.09)	66.71 (54.78 to 79.41)	61.64 (50.43 to 74.08)	-45.3 (-56 to -30.7)	-45.9 (-59.5 to -26.7)	-43.3 (-59 to -20.2)
	DALYs	2237.24 (1859.84 to 2657.76)	2343.4 (1890.65 to 2885.16)	2104.54 (1653.68 to 2673.95)	1264.45 (1116.64 to 1433.91)	1283.25 (1083.9 to 1497.36)	1243.61 (1039.5 to 1475.54)	-43.5 (-54 to -29.7)	-45.2 (-57.9 to -27.5)	-40.9 (-56.3 to -19.6)
	YLLs	1993.8 (1628.69 to 2419.82)	2058.13 (1614.62 to 2582.64)	1903.8 (1440.86 to 2499.88)	1054.95 (917.3 to 1216.16)	1042.1 (860.47 to 1252.65)	1066.49 (868.49 to 1286.05)	-47.1 (-58.1 to -31.7)	-49.4 (-62.8 to -29.6)	-44 (-60.4 to -20.5)
	YLDs	243.44 (174.45 to 317.38)	285.27 (205.92 to 373.31)	200.74 (144.52 to 265.49)	209.5 (149.24 to 269.02)	241.16 (171.66 to 313.65)	177.12 (126.26 to 230.09)	-13.9 (-19.6 to -7.5)	-15.5 (-23 to -7.1)	-11.8 (-19.4 to -2)
North Khorasan	Incidence	181.4 (159.25 to 209.27)	184.44 (160.65 to 211.54)	178.47 (155.26 to 208.12)	151.52 (131.81 to 177.02)	163.08 (140.81 to 191.1)	140.71 (121.69 to 163.81)	-16.5 (-20.4 to -12.1)	-11.6 (-17.5 to -4.3)	-21.2 (-25.8 to -15.9)
	Prevalence	1511.05 (1323.77 to 1732.91)	1702.47 (1477.92 to 1944.53)	1327.36 (1146.53 to 1545.9)	1329.74 (1164.09 to 1506.17)	1489.04 (1283.71 to 1698.51)	1171.62 (1019.13 to 1330.53)	-12 (-17.4 to -6.2)	-12.5 (-20.1 to -4.1)	-11.7 (-18.8 to -2.9)
	Deaths	143.96 (114.33 to 173.99)	143.1 (104.75 to 183.9)	143.22 (110.04 to 189.6)	71.83 (62.25 to 82.6)	77.78 (65.13 to 92.07)	67.46 (56.72 to 80.47)	-50.1 (-60.1 to -35.9)	-45.6 (-59.1 to -23.6)	-52.9 (-65.3 to -35.7)
	DALYs	2893.81 (2397.91 to 3484.78)	2888.64 (2278.22 to 3635.66)	2875.38 (2249.73 to 3764.12)	1438.43 (1273.79 to 1640.43)	1518.57 (1306.58 to 1757.33)	1373.04 (1169.92 to 1638.89)	-50.3 (-59.5 to -39.3)	-47.4 (-59.4 to -30.6)	-52.2 (-64.9 to -35.9)
	YLLs	2660.31 (2165.06 to 3252.13)	2608.92 (1990.15 to 3323.61)	2685.49 (2053.22 to 3576.32)	1230.71 (1078.14 to 1422.44)	1271.23 (1083.25 to 1511.2)	1204.77 (1013.75 to 1453.64)	-53.7 (-63.4 to -42.1)	-51.3 (-63.8 to -33.3)	-55.1 (-67.8 to -38.4)
	YLDs	233.5 (168.48 to 300.38)	279.72 (202.62 to 357.3)	189.89 (135.11 to 249.73)	207.72 (149.04 to 270.01)	247.34 (176.79 to 320.71)	168.27 (119.67 to 218.11)	-11 (-17.1 to -4.5)	-11.6 (-19.7 to -2.2)	-11.4 (-19.4 to -1.7)

Province	Measure	Age-standardized rate (per 100,000)						% Change (1990 to 2019)		
		1990			2019					
		Both	Female	Male	Both	Female	Male	Both	Female	Male
Qazvin	Incidence	156.95 (138.21 to 179.48)	159.26 (140.05 to 181.58)	156.04 (136.37 to 179.13)	140.26 (122.28 to 161.39)	142.92 (124.11 to 166.09)	136.97 (119.36 to 157.81)	-10.6 (-14.4 to -6.6)	-10.3 (-15.2 to -4.6)	-12.2 (-17.8 to -7)
	Prevalence	1297.98 (1142.25 to 1464.77)	1431.05 (1261.41 to 1610.05)	1165.38 (1013.81 to 1329.19)	1236.38 (1087.45 to 1400.36)	1308.42 (1141.48 to 1491.25)	1160.84 (1020.46 to 1328.78)	-4.7 (-10.5 to 0.9)	-8.6 (-15.7 to -1)	-0.4 (-8.4 to 8.1)
	Deaths	112.07 (86.99 to 137.67)	108.88 (78.67 to 138.39)	115.9 (85.93 to 153.32)	78.07 (65.2 to 89.15)	67.09 (52.93 to 79.42)	90 (72.17 to 104.55)	-30.3 (-45.7 to -8.2)	-38.4 (-54.6 to -12.9)	-22.4 (-44.4 to 6.4)
	DALYs	2192.44 (1763.12 to 2669.5)	2171.5 (1664.34 to 2704.04)	2211.07 (1666.81 to 2907.81)	1423.15 (1233.66 to 1606.73)	1230.24 (1030.97 to 1428.39)	1627.95 (1374.91 to 1881.8)	-35.1 (-49.8 to -18)	-43.3 (-57.6 to -24.6)	-26.4 (-47.7 to 0.3)
	YLLs	1990.36 (1582.15 to 2483.49)	1935.57 (1427.76 to 2484.71)	2042.52 (1516.53 to 2741.43)	1227.87 (1036.97 to 1403.76)	1010.07 (816.79 to 1193.78)	1458.78 (1202.2 to 1713.41)	-38.3 (-53.6 to -20)	-47.8 (-62.4 to -27.7)	-28.6 (-50.6 to 0.3)
	YLDs	202.07 (145.66 to 260.17)	235.93 (169.84 to 306.62)	168.56 (120.88 to 218.36)	195.28 (139.64 to 252.34)	220.17 (157.97 to 284.66)	169.17 (120.28 to 218.89)	-3.4 (-9.5 to 2.8)	-6.7 (-15.3 to 1.9)	0.4 (-8.5 to 10)
Qom	Incidence	160.14 (139 to 185.96)	158.23 (135.82 to 183.76)	162.69 (139.39 to 189.62)	116.88 (101.25 to 135.1)	122.76 (105.88 to 143.3)	112.27 (96.18 to 130.17)	-27 (-30 to -23.6)	-22.4 (-27.3 to -17.1)	-31 (-35.1 to -26.5)
	Prevalence	1474.91 (1284.27 to 1679.69)	1621.09 (1415.13 to 1852.24)	1334.56 (1138.07 to 1547.64)	1143.89 (1002.08 to 1316.29)	1263.12 (1094.15 to 1452.01)	1036.45 (899.94 to 1204.44)	-22.4 (-26.9 to -17)	-22.1 (-28.5 to -14.5)	-22.3 (-28.6 to -15.4)
	Deaths	151.8 (116.34 to 184.38)	157.4 (114.54 to 199.92)	144.52 (102.33 to 187.32)	62.17 (52.68 to 70.66)	72.19 (59.41 to 83.21)	56.18 (45.8 to 67.03)	-59 (-68 to -45.5)	-54.1 (-65.7 to -35.4)	-61.1 (-71.6 to -44.8)
	DALYs	2776.5 (2156.09 to 3401.09)	2819.92 (2077.24 to 3605.05)	2711.22 (1967.94 to 3547.48)	1163.25 (1018.13 to 1305.45)	1252.77 (1067.89 to 1437.62)	1104.98 (924.07 to 1303.72)	-58.1 (-67 to -45.9)	-55.6 (-66.2 to -38.3)	-59.2 (-70.1 to -42.7)
	YLLs	2547.88 (1953.82 to 3187.38)	2552.79 (1837.18 to 3340)	2519.84 (1784.97 to 3362.44)	983.68 (849.56 to 1114.36)	1041.02 (870.14 to 1210.67)	954.71 (779.17 to 1144.96)	-61.4 (-70.5 to -48.9)	-59.2 (-70.4 to -41.2)	-62.1 (-73 to -44.7)
	YLDs	228.62 (163.78 to 296.27)	267.13 (190.03 to 344.67)	191.38 (136.04 to 250.24)	179.57 (127.47 to 232.72)	211.75 (151.25 to 274.18)	150.28 (106.66 to 197.29)	-21.5 (-26.6 to -15.7)	-20.7 (-27.9 to -12.4)	-21.5 (-28.7 to -13.6)

Province	Measure	Age-standardized rate (per 100,000)						% Change (1990 to 2019)		
		1990			2019					
		Both	Female	Male	Both	Female	Male	Both	Female	Male
Semnan	Incidence	182.2 (159.22 to 211.33)	179.51 (155.62 to 208.09)	187.04 (162.11 to 216.83)	143.97 (124.33 to 166.41)	149.15 (127.79 to 173.27)	137.95 (118.6 to 160.27)	-21 (-24.6 to -17.4)	-16.9 (-22.3 to -11.3)	-26.2 (-30.5 to -21.1)
	Prevalence	1571.1 (1377.82 to 1785.32)	1675.92 (1471.91 to 1916.29)	1459.57 (1261.64 to 1671.26)	1328.31 (1161.3 to 1508.93)	1416.91 (1235.42 to 1597.05)	1234.86 (1059.19 to 1422.5)	-15.5 (-20.9 to -9.3)	-15.5 (-22.2 to -7.5)	-15.4 (-22.6 to -7.9)
	Deaths	127.59 (100.62 to 153.2)	119.19 (86.47 to 151.41)	137.75 (102.78 to 178.12)	73.89 (62.57 to 84.54)	64.52 (51.59 to 76.52)	84.07 (70.54 to 96.62)	-42.1 (-52.9 to -25.7)	-45.9 (-60 to -23.4)	-39 (-54.2 to -19.9)
	DALYs	2471.33 (1994.51 to 2966.43)	2309.89 (1756.32 to 2928.32)	2640.82 (2019.08 to 3374.09)	1325.42 (1153.31 to 1497.53)	1166.9 (978.72 to 1362.19)	1491.7 (1288.31 to 1702.6)	-46.4 (-56.4 to -33)	-49.5 (-61.3 to -32.1)	-43.5 (-56.9 to -25.3)
	YLLs	2228.55 (1759.76 to 2711.58)	2034.27 (1482.01 to 2671.09)	2432.59 (1818.46 to 3148.51)	1116.29 (959.39 to 1279.34)	929.34 (752.21 to 1109.29)	1312.39 (1114.69 to 1522.51)	-49.9 (-60.1 to -35.3)	-54.3 (-67.1 to -35.4)	-46 (-59.5 to -26.8)
	YLDs	242.78 (170.59 to 312.57)	275.62 (195.42 to 357.22)	208.23 (146.53 to 271.55)	209.13 (151.07 to 266.55)	237.57 (172.72 to 306.44)	179.31 (126.4 to 231.79)	-13.9 (-20 to -7)	-13.8 (-21.3 to -4.8)	-13.9 (-21.8 to -5.5)
Sistan and Baluchistan	Incidence	153.96 (135.02 to 176.88)	152.87 (133.63 to 176.44)	155.22 (136.1 to 178.86)	135.3 (119.17 to 155.07)	137.84 (120.04 to 158.6)	133.01 (116.73 to 153.19)	-12.1 (-15.6 to -8.5)	-9.8 (-15.6 to -4.1)	-14.3 (-19.4 to -9.1)
	Prevalence	1259.38 (1110.75 to 1423.97)	1375.4 (1219.81 to 1540.71)	1156.03 (1004.17 to 1339.78)	1144.63 (1018.7 to 1288.34)	1216.96 (1077.5 to 1365.68)	1073.17 (940.7 to 1230.79)	-9.1 (-14.2 to -3.2)	-11.5 (-18.2 to -4.6)	-7.2 (-14.6 to 1)
	Deaths	112.05 (86.07 to 137.2)	99.56 (71.34 to 131.26)	123.75 (90.38 to 159.75)	63.49 (52.63 to 75.55)	58.62 (46.21 to 71.99)	68.47 (53.73 to 86.35)	-43.3 (-57.2 to -26.4)	-41.1 (-58.6 to -12.9)	-44.7 (-61.2 to -21.9)
	DALYs	2260.13 (1681.88 to 2791.08)	2062.97 (1528.32 to 2705.21)	2421.16 (1704.12 to 3197.92)	1426.64 (1209.16 to 1670.32)	1321.86 (1085.74 to 1597.02)	1533.04 (1241.5 to 1893.32)	-36.9 (-52.4 to -15.4)	-35.9 (-53.4 to -10)	-36.7 (-55.3 to -6.8)
	YLLs	2066.08 (1494.96 to 2610.24)	1836.85 (1306.34 to 2452.18)	2254.48 (1530.85 to 3034.37)	1248.69 (1037.07 to 1479.12)	1120.3 (884.86 to 1382.62)	1378.36 (1082.68 to 1726.03)	-39.6 (-55.3 to -16.3)	-39 (-57.6 to -9.9)	-38.9 (-58 to -6.8)
	YLDs	194.05 (138.77 to 248.18)	226.12 (162.26 to 288.1)	166.68 (119.18 to 216.94)	177.95 (128.14 to 228.8)	201.56 (145.87 to 258.55)	154.68 (110.77 to 201.76)	-8.3 (-14.1 to -2)	-10.9 (-18.2 to -3.3)	-7.2 (-15.3 to 2.3)

Province	Measure	Age-standardized rate (per 100,000)						% Change (1990 to 2019)		
		1990			2019					
		Both	Female	Male	Both	Female	Male	Both	Female	Male
South Khorasan	Incidence	161.03 (141.2 to 184.45)	162.09 (141.14 to 186.78)	160.3 (139.89 to 184.5)	134.77 (117.39 to 155.55)	139.79 (121.21 to 161.82)	129.63 (112.65 to 149.23)	-16.3 (-20.1 to -13)	-13.8 (-18.7 to -8.7)	-19.1 (-24.2 to -14.1)
	Prevalence	1297.61 (1145.11 to 1475.22)	1446.16 (1264.86 to 1645.86)	1157.91 (1008.68 to 1326.92)	1169.24 (1036.06 to 1322.33)	1247.29 (1092.53 to 1410.19)	1088.11 (959.94 to 1243.86)	-9.9 (-15.1 to -4.2)	-13.8 (-21 to -6.1)	-6 (-13.1 to 1.1)
	Deaths	105.22 (85.27 to 126.01)	102.02 (76.79 to 129.12)	108.35 (85.91 to 137.32)	59.42 (50.3 to 67.64)	56.82 (46.4 to 67.42)	63.09 (53.32 to 74.74)	-43.5 (-54 to -28.4)	-44.3 (-57.4 to -23.6)	-41.8 (-56 to -23.3)
	DALYs	2036.18 (1719.25 to 2464.32)	1997.19 (1586.44 to 2463.8)	2071.84 (1644.64 to 2592)	1113.38 (974.93 to 1257.56)	1084.34 (920.55 to 1248.82)	1150.32 (988.48 to 1369.84)	-45.3 (-55 to -34)	-45.7 (-57.4 to -30.2)	-44.5 (-56.7 to -28.4)
	YLLs	1834.86 (1529.61 to 2275.36)	1758.99 (1370.53 to 2252.8)	1904.92 (1484.49 to 2437.42)	930.82 (805.89 to 1069.54)	877.53 (725.07 to 1041.54)	993.05 (832.14 to 1216.08)	-49.3 (-59 to -37.5)	-50.1 (-62.3 to -32.9)	-47.9 (-60.3 to -31.1)
	YLDs	201.32 (144.43 to 259.45)	238.2 (169.73 to 307.78)	166.92 (120.08 to 216.43)	182.56 (131.49 to 234.69)	206.81 (148.78 to 267.69)	157.27 (111.2 to 203.18)	-9.3 (-15.1 to -2.6)	-13.2 (-21 to -4.6)	-5.8 (-13.6 to 3.4)
Tehran	Incidence	146.08 (127.58 to 167.87)	141.05 (122.97 to 163.31)	151.63 (131.31 to 174.09)	122.91 (107.02 to 142.65)	124.75 (107.94 to 145.53)	121.42 (105.38 to 140.8)	-15.9 (-19.5 to -11.9)	-11.6 (-17.1 to -6)	-19.9 (-25 to -14.7)
	Prevalence	1267.38 (1118.7 to 1433.05)	1344.74 (1177.95 to 1514.54)	1190.11 (1030.1 to 1370.16)	1084.88 (961.43 to 1221.11)	1149.97 (1011.38 to 1295.02)	1022.43 (895.14 to 1160.29)	-14.4 (-18.9 to -9.1)	-14.5 (-20.8 to -7.5)	-14.1 (-21.7 to -6.5)
	Deaths	66.93 (52.3 to 83.97)	68.57 (50.48 to 90.62)	63.96 (48.18 to 83.73)	37.78 (31.35 to 44.36)	42.69 (34.06 to 51.89)	33.73 (26.09 to 42)	-43.6 (-57.7 to -25.8)	-37.7 (-57.1 to -12.3)	-47.3 (-62.7 to -25.6)
	DALYs	1336.25 (1086.32 to 1608.47)	1309.57 (1021.3 to 1656.05)	1340.99 (1036.72 to 1720.23)	751.86 (651.39 to 874.34)	792.97 (664.67 to 943.81)	717.94 (590.11 to 863.8)	-43.7 (-54.9 to -29.9)	-39.4 (-54.8 to -19.9)	-46.5 (-60.4 to -27.6)
	YLLs	1139.48 (909.26 to 1406.91)	1087.55 (810.6 to 1423.5)	1169.66 (875.59 to 1550.23)	582.51 (495.82 to 682.58)	601.7 (485.78 to 732.14)	569.91 (451.74 to 715.47)	-48.9 (-61.1 to -33)	-44.7 (-61.4 to -22.2)	-51.3 (-66.2 to -30)
	YLDs	196.77 (142.24 to 253.12)	222.01 (161.69 to 282.79)	171.32 (121.65 to 225.19)	169.34 (121.15 to 217.81)	191.27 (137.61 to 244.86)	148.03 (106.12 to 191.35)	-13.9 (-18.8 to -8.2)	-13.8 (-21 to -5.5)	-13.6 (-22 to -5.5)

Province	Measure	Age-standardized rate (per 100,000)						% Change (1990 to 2019)		
		1990			2019					
		Both	Female	Male	Both	Female	Male	Both	Female	Male
West Azarbayejan	Incidence	172.04 (149.92 to 197.51)	175.22 (152.84 to 203.25)	169.36 (148.23 to 195.75)	147.28 (127.66 to 169.74)	154.56 (133.74 to 178.79)	139.37 (120.8 to 162.83)	-14.4 (-18.1 to -10.6)	-11.8 (-17.4 to -5.9)	-17.7 (-22.6 to -12.4)
	Prevalence	1489.66 (1310.24 to 1704.39)	1680.62 (1469.38 to 1919.69)	1308.2 (1121.69 to 1528.12)	1312.88 (1155.16 to 1497.42)	1435.71 (1252.42 to 1638.53)	1183.76 (1035.05 to 1372.27)	-11.9 (-17.1 to -5.6)	-14.6 (-21.4 to -6.7)	-9.5 (-17.4 to -0.8)
	Deaths	141.02 (116.3 to 166)	141.59 (109.37 to 174.84)	138.96 (109.91 to 171.4)	87.33 (75.35 to 98.17)	89.44 (74.4 to 103.7)	85.05 (70.89 to 100.08)	-38.1 (-49.9 to -22.3)	-36.8 (-50.9 to -13.7)	-38.8 (-53.5 to -20.1)
	DALYs	2584.12 (2170.81 to 3050.47)	2595.01 (2073.51 to 3188.77)	2557.66 (2048.01 to 3167.72)	1514.35 (1324.37 to 1696.45)	1510.96 (1280.01 to 1738.8)	1514.17 (1275.09 to 1781.25)	-41.4 (-51.9 to -27.9)	-41.8 (-54.6 to -24.8)	-40.8 (-55.1 to -22.8)
	YLLs	2351.77 (1942.08 to 2803.44)	2316.81 (1800.9 to 2907.1)	2368.42 (1840.48 to 2971.17)	1307.38 (1131.2 to 1479.44)	1270.92 (1064.32 to 1493.89)	1342.08 (1106.29 to 1601.6)	-44.4 (-55.7 to -30.3)	-45.1 (-58.3 to -26.3)	-43.3 (-58.3 to -24.3)
	YLDs	232.35 (168.72 to 298.66)	278.19 (200.74 to 357.39)	189.24 (133.63 to 250.42)	206.97 (148.97 to 270.43)	240.04 (173.23 to 310.26)	172.1 (123.1 to 223.42)	-10.9 (-16.7 to -4.1)	-13.7 (-21.8 to -4.9)	-9.1 (-18.1 to 0.6)
Yazd	Incidence	162.78 (141.96 to 186.75)	162.68 (142.17 to 187.36)	166.59 (145.8 to 192.55)	135.13 (117.63 to 155.43)	140.31 (122.29 to 162.38)	129.3 (112.03 to 148.87)	-17 (-20.9 to -12.9)	-13.7 (-19.3 to -7.5)	-22.4 (-26.7 to -17.7)
	Prevalence	1396.27 (1227.77 to 1601.92)	1516.79 (1326.88 to 1734.26)	1271.54 (1109.57 to 1475.63)	1215.28 (1068.97 to 1370.47)	1308.8 (1142.35 to 1484.07)	1121.17 (985.1 to 1280.92)	-13 (-18.1 to -8)	-13.7 (-20.3 to -6.4)	-11.8 (-19.2 to -4.2)
	Deaths	134.97 (106.44 to 163.27)	134.43 (97.56 to 168.59)	133.29 (100.59 to 169.9)	76.7 (64.2 to 89.18)	76.23 (61.61 to 91.78)	77.26 (61.97 to 93.33)	-43.2 (-55.5 to -26.2)	-43.3 (-58.4 to -21.5)	-42 (-57.4 to -20)
	DALYs	2424.2 (1971.89 to 2917.62)	2414.12 (1852.98 to 3014.97)	2416.67 (1847.54 to 3088.29)	1312.69 (1137.98 to 1510.05)	1288.28 (1069.04 to 1524.5)	1332.57 (1094.06 to 1599.36)	-45.9 (-56.8 to -31.8)	-46.6 (-59.5 to -29.2)	-44.9 (-58.8 to -23.9)
	YLLs	2205.92 (1754.91 to 2690.12)	2162.88 (1604.32 to 2749.34)	2233.54 (1676.59 to 2903.19)	1121.68 (949.98 to 1309.63)	1068.65 (868.38 to 1306.88)	1170.11 (934.62 to 1429.52)	-49.2 (-61.1 to -34.1)	-50.6 (-64.2 to -32)	-47.6 (-61.9 to -24.8)
	YLDs	218.27 (157.11 to 285.37)	251.24 (181.77 to 327.89)	183.13 (129.75 to 242.47)	191.01 (137.28 to 245.18)	219.63 (158.55 to 281.93)	162.46 (113.8 to 211.93)	-12.5 (-18.3 to -6.8)	-12.6 (-20 to -4.2)	-11.3 (-19.4 to -2.6)

Province	Measure	Age-standardized rate (per 100,000)						% Change (1990 to 2019)		
		1990			2019					
		Both	Female	Male	Both	Female	Male	Both	Female	Male
Zanjan	Incidence	169.08 (148.01 to 195.67)	166.16 (145.36 to 192.41)	173.46 (150.24 to 199.93)	142.47 (124.19 to 164.58)	147.4 (127.47 to 170.99)	137.04 (119.36 to 159)	-15.7 (-19.4 to -12)	-11.3 (-16.5 to -4.9)	-21 (-25.9 to -16.2)
	Prevalence	1470.43 (1291.1 to 1669.57)	1566.98 (1377.47 to 1784.81)	1368.63 (1181.32 to 1579.04)	1267.54 (1110.49 to 1449.99)	1354.2 (1188.46 to 1555.54)	1177.02 (1016.79 to 1352.22)	-13.8 (-19 to -8.1)	-13.6 (-20.7 to -6.3)	-14 (-21.4 to -5.2)
	Deaths	110.31 (89.14 to 133.44)	99.36 (75.15 to 124.01)	122.75 (90.01 to 154.28)	69.81 (59.16 to 77.9)	59.63 (47.88 to 69.42)	81.23 (67.1 to 94.21)	-36.7 (-49.3 to -21.4)	-40 (-54.4 to -19.2)	-33.8 (-50.2 to -13.6)
	DALYs	2211.23 (1837.14 to 2660.12)	2008.19 (1618.71 to 2464.87)	2406.48 (1912.93 to 3015)	1296 (1145.45 to 1440.14)	1126.1 (956.1 to 1290.28)	1480.21 (1274.81 to 1700.26)	-41.4 (-52.7 to -29.3)	-43.9 (-56.2 to -28.7)	-38.5 (-52.7 to -20)
	YLLs	1983.36 (1616.01 to 2426.82)	1749.74 (1379.7 to 2223.13)	2209.34 (1723.8 to 2817.95)	1096.95 (953.68 to 1220.94)	899.73 (747.59 to 1045.66)	1309.98 (1108.43 to 1530.49)	-44.7 (-56.7 to -31.7)	-48.6 (-62 to -31.9)	-40.7 (-55.8 to -21)
	YLDs	227.87 (165.86 to 294.6)	258.45 (186.13 to 337.37)	197.14 (142.1 to 259.68)	199.05 (141.37 to 255.93)	226.38 (162.8 to 291.05)	170.23 (119.42 to 219.88)	-12.7 (-18.4 to -6.6)	-12.4 (-20 to -4.3)	-13.7 (-21.8 to -3.9)

Data in parentheses are 95% Uncertainty Intervals (95% UIs)
