# Supplementary material for: National and subnational burden of stroke in Iran from 1990 to 2019
Source: Ann Clin Transl Neurol. 2022 Apr 8;9(5):669–83. doi: 10.1002/acn3.51547 (PMC9082377; doi:10.1002/acn3.51547)
Supplement: Supplementary file 4 — Supplementary Table S3 Decomposition analysis of stroke new cases between 1990 and 2019 by sex at national and subnational levels. [file ACN3-9-669-s003.pdf]

Supplementary Table 3. Decomposition analysis of stroke new cases between 1990 and 2019 by sex at national and sub-national levels

Location		Sex	New cases		Expected new cases in 2019		% 1990 - 2019 new cases change cause			% 1990 - 2019 new cases overall change
			1990	2019	Population growth	Population growth + Aging	Population growth	Age structure change	Incidence rate change	
**Iran (Islamic Republic of)**		Female	23725	52562	34389	62938	44.9%	120.3%	-43.7%	121.5%
		Male	24549	50216	35128	62645	43.1%	112.1%	-50.6%	104.6%
		Both	48274	102778	69515	125436	44%	115.8%	-46.9%	112.9%
**Subnational**	Alborz	Female	574	1817	1142	2300	99%	201.8%	-84.1%	216.7%
		Male	573	1822	1100	2238	92%	198.6%	-72.5%	218.1%
	Ardebil	Female	440	952	488	988	10.9%	113.6%	-8.1%	116.4%
		Male	467	857	517	928	10.8%	88.1%	-15.1%	83.8%
	Bushehr	Female	287	725	472	827	64.7%	123.8%	-35.6%	152.9%
		Male	294	673	523	858	78%	113.8%	-62.8%	129%
	Chahar Mahaal and Bakhtiari	Female	258	586	351	675	36%	125.8%	-34.5%	127.3%
		Male	279	559	376	661	34.8%	102.3%	-36.6%	100.5%
	East Azarbayejan	Female	1582	2944	1869	3627	18.1%	111.1%	-43.2%	86%
		Male	1688	2787	1995	3577	18.2%	93.7%	-46.7%	65.1%
	Fars	Female	1454	3268	1985	3812	36.5%	125.7%	-37.4%	124.8%
		Male	1436	3125	1944	3657	35.4%	119.3%	-37%	117.7%
	Gilan	Female	1246	2458	1392	2850	11.7%	117%	-31.4%	97.3%
		Male	1160	2267	1292	2730	11.4%	123.9%	-39.9%	95.5%
	Golestan	Female	601	1416	867	1581	44.3%	118.9%	-27.6%	135.6%
		Male	590	1256	846	1426	43.5%	98.2%	-28.8%	113%
	Hamadan	Female	747	1370	786	1558	5.1%	103.3%	-25.1%	83.3%
		Male	752	1272	766	1478	1.8%	94.6%	-27.4%	69.1%
	Hormozgan	Female	365	910	749	1114	105.1%	99.8%	-55.7%	149.2%
		Male	407	904	833	1119	104.9%	70.3%	-53%	122.2%
	Ilam	Female	124	319	166	339	33.7%	139.2%	-16.5%	156.4%
		Male	151	302	198	355	30.4%	103.7%	-35%	99.2%
	Isfahan	Female	1449	2992	2025	3763	39.7%	120%	-53.3%	106.4%
		Male	1515	3039	2040	4044	34.6%	132.3%	-66.3%	100.6%
	Kerman	Female	670	1616	1179	1939	76%	113.6%	-48.3%	141.3%
		Male	681	1541	1230	1918	80.5%	101.1%	-55.4%	126.3%

Supplementary Table 3. Decomposition analysis of stroke new cases between 1990 and 2019 by sex at national and sub-national levels										
**Location**		**Sex**	**New cases**		**Expected new cases in 2019**		**% 1990 - 2019 new cases change cause**			**% 1990 - 2019 new cases overall change**
			**1990**	**2019**	**Population growth**	**Population growth + Aging**	**Populatio n growth**	**Age structure change**	**Incidence rate change**	
	Kermanshah	Female	707	1482	840	1753	18.7%	129.2%	-38.4%	109.5%
		Male	803	1372	916	1725	14.1%	100.8%	-44%	70.9%
	Khorasan-e-Razavi	Female	2157	3925	3056	5263	41.7%	102.3%	-62%	81.9%
		Male	2278	3690	3214	5090	41.1%	82.4%	-61.5%	62%
	Khuzestan	Female	1264	3083	1936	3331	53.2%	110.3%	-19.6%	143.9%
		Male	1256	2905	1899	3244	51.3%	107.1%	-27%	131.3%
	Kohgiluyeh and Boyer-Ahmad	Female	154	375	233	417	51.9%	119.4%	-27.3%	144%
		Male	170	378	259	460	52%	118.2%	-48.4%	121.8%
	Kurdistan	Female	480	1012	644	1221	34.2%	120.3%	-43.6%	110.9%
		Male	533	997	712	1218	33.5%	94.8%	-41.4%	86.9%
	Lorestan	Female	566	1136	654	1355	15.5%	123.9%	-38.7%	100.8%
		Male	654	1102	740	1344	13.2%	92.2%	-36.9%	68.5%
	Markazi	Female	579	1092	687	1345	18.5%	113.5%	-43.7%	88.4%
		Male	585	1014	702	1336	19.9%	108.4%	-55%	73.2%
	Mazandaran	Female	1223	2868	1606	3401	31.3%	146.8%	-43.6%	134.4%
		Male	1144	2574	1507	3158	31.6%	144.3%	-51.1%	124.9%
	North Khorasan	Female	279	595	384	695	37.6%	111.3%	-35.8%	113.1%
		Male	283	520	389	656	37.3%	94.1%	-47.8%	83.6%
	Qazvin	Female	371	822	520	961	40.4%	118.9%	-37.5%	121.8%
		Male	364	777	508	882	39.3%	102.5%	-28.8%	113.1%
	Qom	Female	266	622	495	844	85.8%	131%	-83.6%	133.3%
		Male	276	636	504	913	82.4%	148%	-100.3%	130.1%
	Semnan	Female	261	513	410	637	57.1%	87.3%	-47.7%	96.7%
		Male	266	465	406	632	52.9%	85.1%	-62.9%	75.1%
	Sistan and Baluchistan	Female	465	1109	940	1248	102%	66.2%	-29.9%	138.4%
		Male	528	1080	1066	1192	101.9%	23.7%	-21.2%	104.4%
	South Khorasan	Female	298	520	370	610	24.1%	80.3%	-30.3%	74.1%
		Male	314	459	390	568	24.4%	56.5%	-34.8%	46.1%
	Tehran	Female	3215	8457	5380	9885	67.3%	140.1%	-44.4%	163%

Supplementary Table 3. Decomposition analysis of stroke new cases between 1990 and 2019 by sex at national and sub-national levels										
**Location**		**Sex**	**New cases**		**Expected new cases in 2019**		**% 1990 - 2019 new cases change cause**			**% 1990 - 2019 new cases overall change**
			**1990**	**2019**	**Population growth**	**Population growth + Aging**	**Population growth**	**Age structure change**	**Incidence rate change**	
		Male	3453	8622	5543	10873	60.5%	154.4%	-65.2%	149.7%
	West Azarbayejan	Female	963	2160	1413	2566	46.8%	119.8%	-42.2%	124.4%
		Male	961	1919	1409	2322	46.5%	95%	-41.9%	99.7%
	Yazd	Female	310	665	527	792	70.3%	85.5%	-40.9%	114.8%
		Male	293	633	492	807	68.1%	107.4%	-59.3%	116.3%
	Zanjan	Female	369	753	450	876	22.1%	115.3%	-33.3%	104.1%
		Male	393	669	476	846	21%	94.1%	-45.1%	70%
