# Supplementary material for: National and subnational burden of stroke in Iran from 1990 to 2019
Source: Ann Clin Transl Neurol. 2022 Apr 8;9(5):669–83. doi: 10.1002/acn3.51547 (PMC9082377; doi:10.1002/acn3.51547)
Supplement: Supplementary file 5 — Supplementary Table S4 National and subnational age‐standardized rate of attributed deaths, DALYs, YLLs, and YLDs due to all risk factors in 1990 and 2019, with percentage change by sex. [file ACN3-9-669-s001.pdf]

Supplementary Table 4. National and sub-national age-standardized rate of attributed deaths, DALYs, YLLs, and YLDs due to all risk factors in 1990 and 2019, with percentage change by sex

Province	Measure	Attributed age-standardized rate (per 100,000)						% Change (1990 to 2019)		
		1990			2019					
		Both	Female	Male	Both	Female	Male	Both	Female	Male
Iran	Deaths	97 (80.7 to 111.5)	95.4 (76.2 to 111.6)	97.2 (82.1 to 112.5)	54.7 (47.4 to 61.1)	56 (47.8 to 63.5)	53.7 (46.6 to 60)	-43.6 (-49.3 to -33.2)	-41.3 (-48.2 to -26.3)	-44.8 (-53.1 to -35.5)
	DALYs	1911.6 (1660.7 to 2129.8)	1859.3 (1578.8 to 2094.4)	1944.9 (1683.9 to 2197.4)	1072.1 (972.6 to 1165.2)	1058.3 (935.1 to 1166)	1088.5 (992 to 1187.8)	-43.9 (-49.4 to -35.4)	-43.1 (-49.2 to -31.6)	-44 (-51.2 to -35.5)
	YLLs	1725.3 (1487.2 to 1941)	1641.7 (1371.4 to 1864.8)	1788.7 (1539 to 2046.3)	905.8 (819.4 to 984)	868.2 (766.8 to 959.1)	946 (855.1 to 1033.2)	-47.5 (-53.4 to -38.7)	-47.1 (-53.4 to -34.8)	-47.1 (-54.6 to -38.2)
	YLDs	186.3 (133.9 to 241.9)	217.6 (155.9 to 280.2)	156.2 (111.1 to 204.2)	166.3 (118.7 to 213.7)	190.1 (136.4 to 245.1)	142.6 (101 to 185.2)	-10.7 (-14.1 to -7.4)	-12.6 (-16.1 to -8.7)	-8.7 (-13 to -4.4)
Alborz	Deaths	102.12 (77.61 to 127.47)	109.85 (79.01 to 139.55)	93.97 (65.97 to 126.41)	53.74 (45.5 to 63.5)	61.08 (49.93 to 73.85)	49.9 (39.61 to 61.62)	-47.4 (-59.9 to -29.2)	-44.4 (-59.6 to -19.2)	-46.9 (-62.9 to -24.9)
	DALYs	1878.72 (1485.61 to 2351.82)	1986.18 (1456.07 to 2534.38)	1765.65 (1256.26 to 2378.98)	1017.65 (878.85 to 1168.38)	1066.16 (898.99 to 1244.52)	996.88 (819.16 to 1202.29)	-45.8 (-58.1 to -26.3)	-46.3 (-59.8 to -24.6)	-43.5 (-59.9 to -20.7)
	YLLs	1677.78 (1276.35 to 2137.83)	1746.52 (1250 to 2285.48)	1600.17 (1108.14 to 2185.09)	838.73 (718.95 to 982.2)	858.93 (708.28 to 1028.44)	844.21 (677.91 to 1044.18)	-50 (-62.5 to -29.6)	-50.8 (-65 to -26.6)	-47.2 (-64 to -21.9)
	YLDs	200.94 (144.14 to 260.27)	239.66 (172.07 to 311.15)	165.48 (117.72 to 217.02)	178.93 (126.78 to 232.45)	207.23 (148.74 to 270.98)	152.67 (107.45 to 200.75)	-11 (-17.2 to -4.2)	-13.5 (-22 to -3.3)	-7.7 (-16.9 to 1.7)
Ardebil	Deaths	90.16 (70.07 to 111.11)	84.76 (62.88 to 110.06)	95.42 (70.9 to 124.62)	63.12 (53.84 to 71.97)	54.83 (45.41 to 64.64)	72.02 (60.43 to 83.42)	-30 (-45.7 to -9.1)	-35.3 (-51.4 to -9)	-24.5 (-43.8 to 2.5)
	DALYs	1878.53 (1509.62 to 2312.61)	1802.41 (1396.18 to 2266.18)	1945.27 (1477.29 to 2504.38)	1261.93 (1110.67 to 1418.89)	1107 (951.32 to 1275.54)	1425.56 (1225.39 to 1644.5)	-32.8 (-47.5 to -14.9)	-38.6 (-53.1 to -16.9)	-26.7 (-46.1 to -2.8)
	YLLs	1697.61 (1343.81 to 2124.15)	1589.83 (1193.27 to 2067.72)	1792.8 (1324.96 to 2340.96)	1079.7 (944.23 to 1229.34)	898.22 (756.94 to 1047.11)	1270.98 (1071.17 to 1468.52)	-36.4 (-50.8 to -17.2)	-43.5 (-58.7 to -20.5)	-29.1 (-49.2 to -3.3)
	YLDs	180.92	212.58	152.48	182.23	208.78	154.57	0.7 (-6.9 to	-1.8 (-11.4	1.4 (-9.2 to

		(128.94 to 235.66)	(150.86 to 277.23)	(107.94 to 197.61)	(130.72 to 236.32)	(149.63 to 270.5)	(109.62 to 201.25)	9.1)	to 8.4)	13.5)

Province	Measure	Attributed age-standardized rate (per 100,000)						% Change (1990 to 2019)		
		1990			2019					
		Both	Female	Male	Both	Female	Male	Both	Female	Male
Bushehr	Deaths	126.08 (95.79 to 152.64)	118.09 (87.67 to 146.83)	133.56 (90.4 to 167.84)	80.59 (66.05 to 92.23)	73.61 (60.52 to 85.45)	87.9 (68.67 to 103.51)	-36.1 (-48.9 to -20)	-37.7 (-50.8 to -18.8)	-34.2 (-50.6 to -11.9)
	DALYs	2405.08 (1865.53 to 2868.46)	2252.76 (1752.47 to 2766.65)	2544.46 (1835.25 to 3183.75)	1514.91 (1304.17 to 1698.37)	1403.99 (1199.2 to 1605.93)	1631.11 (1333.04 to 1881.88)	-37 (-49.7 to -21.8)	-37.7 (-50.1 to -19.2)	-35.9 (-51.7 to -13.7)
	YLLs	2187.57 (1674.85 to 2635.09)	2005 (1489.86 to 2511.18)	2356.22 (1652.27 to 2998.39)	1317.07 (1110.7 to 1487.43)	1173.35 (988.29 to 1344.6)	1465.67 (1169.82 to 1720.14)	-39.8 (-53 to -23.2)	-41.5 (-54.5 to -21)	-37.8 (-54.2 to -13.9)
	YLDs	217.51 (155.48 to 283.48)	247.76 (175.16 to 319.67)	188.25 (134.67 to 249.3)	197.85 (141.27 to 256.88)	230.65 (163.97 to 300.46)	165.44 (117.76 to 216.62)	-9 (-15.8 to -1.1)	-6.9 (-16.1 to 3.9)	-12.1 (-21 to -2.1)
BakhtiariMahaalChahar	Deaths	83.25 (65.39 to 102.65)	77.32 (56.4 to 102.33)	88.5 (66.48 to 116.74)	49.86 (41.17 to 59.6)	40.83 (31.09 to 51.96)	59.42 (47.48 to 70.7)	-40.1 (-53.8 to -21.8)	-47.2 (-63.1 to -22.5)	-32.9 (-51.7 to -8)
	DALYs	1690.61 (1383.1 to 2063.08)	1573.34 (1191.78 to 2013.7)	1789.11 (1364.89 to 2344.11)	975.27 (836.8 to 1119.8)	821.65 (678.41 to 997.41)	1136.68 (942.33 to 1343.14)	-42.3 (-54.7 to -26.4)	-47.8 (-61.6 to -28.8)	-36.5 (-53.4 to -12.2)
	YLLs	1519.18 (1208.59 to 1894.19)	1376.07 (1015.49 to 1815.85)	1641.93 (1215.36 to 2186.07)	813.41 (685.01 to 954.29)	640.17 (503.51 to 815.56)	995.25 (813.28 to 1200.55)	-46.5 (-59.1 to -29.4)	-53.5 (-68.1 to -32.9)	-39.4 (-56.4 to -13.3)
	YLDs	171.44 (123.46 to 223.65)	197.27 (143.72 to 255.6)	147.17 (103.62 to 192.66)	161.86 (115.99 to 209.61)	181.48 (127.06 to 235.97)	141.43 (101.48 to 185.04)	-5.6 (-12.6 to 1.7)	-8 (-17.1 to 3.3)	-3.9 (-13 to 6.3)
East Azarbayejan	Deaths	121.16 (96.53 to 148.09)	122.61 (88.08 to 158.11)	118.06 (88 to 152.73)	68.83 (57.25 to 85.33)	70.55 (55.68 to 90.42)	67.08 (53.96 to 84.13)	-43.2 (-57 to -22.8)	-42.5 (-58.1 to -16.8)	-43.2 (-60.4 to -17.6)
	DALYs	2211.7 (1782.45 to 2691.27)	2202.73 (1674.64 to 2817.91)	2200.27 (1659.86 to 2856.59)	1233.5 (1049.92 to 1465.69)	1224.69 (1012.61 to 1511.36)	1240.85 (1028.79 to 1514.34)	-44.2 (-57.3 to -26.7)	-44.4 (-59.8 to -24.2)	-43.6 (-59.9 to -18.9)
	YLLs	2003.38 (1576.86 to 2472.13)	1957.79 (1436.75 to 2593.42)	2025.96 (1487.83 to 2683.63)	1050.16 (888.55 to 1284.93)	1016.14 (814.3 to 1290.72)	1082.98 (882.3 to 1347.38)	-47.6 (-60.8 to -28.5)	-48.1 (-63.6 to -26.1)	-46.5 (-62.8 to -19.9)
	YLDs	208.32 (150.04 to 272.61)	244.93 (175.58 to 317.02)	174.3 (122.26 to 228.1)	183.35 (129.39 to 236.3)	208.54 (147.01 to 273.81)	157.87 (112.01 to 204.52)	-12 (-19.1 to -4.4)	-14.9 (-24 to -4.5)	-9.4 (-18.7 to 1.1)

Province	Measure	Attributed age-standardized rate (per 100,000)						% Change (1990 to 2019)		
		1990			2019					
		Both	Female	Male	Both	Female	Male	Both	Female	Male
Fars	Deaths	97.11 (75.49 to 117.35)	95.4 (70.86 to 121.57)	97.08 (71.92 to 124.39)	67.11 (54.82 to 79.12)	68.33 (53.96 to 83.15)	66.45 (51.38 to 83.12)	-30.9 (-46.4 to -12.6)	-28.4 (-46.8 to -2)	-31.6 (-50.5 to -5.3)
	DALYs	1851.43 (1497.81 to 2225.8)	1794.42 (1366.94 to 2261.59)	1885.12 (1421.52 to 2378.54)	1243.23 (1052.07 to 1435.02)	1205.72 (989.39 to 1427.42)	1284.07 (1042.37 to 1574.62)	-32.9 (-47.4 to -15.4)	-32.8 (-49.5 to -9.3)	-31.9 (-51.3 to -5.6)
	YLLs	1666.9 (1338.85 to 2042.46)	1574.92 (1138.71 to 2026.52)	1735.27 (1287.1 to 2223.21)	1071.33 (889.88 to 1255.83)	1008.76 (809.77 to 1226.64)	1136.95 (898.66 to 1428.61)	-35.7 (-51.1 to -16.5)	-35.9 (-54.1 to -9.4)	-34.5 (-54.4 to -6.6)
	YLDs	184.53 (131.28 to 239.02)	219.5 (156.88 to 287.9)	149.85 (106.34 to 194.83)	171.9 (123.33 to 221.08)	196.96 (141.06 to 255.93)	147.12 (104.76 to 192.69)	-6.8 (-14.2 to 0.9)	-10.3 (-19.5 to 0.4)	-1.8 (-12.7 to 9.7)
Gilan	Deaths	120.27 (93.9 to 146.07)	122.01 (92.7 to 153.48)	114.37 (81.64 to 147.41)	68.51 (57.25 to 79.46)	73.6 (58.75 to 87.98)	63.98 (51.54 to 77.59)	-43 (-54.2 to -26.4)	-39.7 (-56.1 to -16.9)	-44.1 (-59.3 to -21.9)
	DALYs	2336.43 (1863.24 to 2818.82)	2344.12 (1828.67 to 2905.67)	2285.02 (1689.05 to 2956.96)	1329.9 (1157.33 to 1512.15)	1354.63 (1127.08 to 1591.78)	1307.92 (1089.5 to 1574.94)	-43.1 (-53.9 to -27.6)	-42.2 (-56.3 to -22.2)	-42.8 (-58.1 to -20.6)
	YLLs	2125.33 (1663.38 to 2612.51)	2095.3 (1575.23 to 2666.76)	2115.12 (1535.9 to 2746.01)	1140.49 (973.16 to 1313.49)	1135.82 (923.13 to 1362.14)	1148.47 (926.93 to 1405.38)	-46.3 (-57.4 to -30.1)	-45.8 (-60.4 to -23.8)	-45.7 (-61.4 to -22.3)
	YLDs	211.11 (149.31 to 275.78)	248.82 (178.78 to 323.88)	169.89 (121.1 to 223.39)	189.41 (135.18 to 245.62)	218.81 (155.43 to 283.94)	159.45 (113.83 to 208.89)	-10.3 (-17.1 to -3.1)	-12.1 (-20.6 to -2.6)	-6.1 (-15.8 to 3.5)
Golestan	Deaths	118.46 (92.9 to 145.19)	115.6 (86.67 to 143.28)	119.26 (88.96 to 153.02)	75.4 (65.01 to 85.55)	71.91 (59.64 to 84.54)	79.42 (65.72 to 94)	-36.3 (-48.8 to -17.9)	-37.8 (-52.6 to -14)	-33.4 (-51.3 to -8.5)
	DALYs	2480.64 (1958.23 to 2978.28)	2428.57 (1856.3 to 2975.94)	2502.31 (1877.44 to 3229.29)	1567.84 (1378.74 to 1752.41)	1499.44 (1284.42 to 1736.68)	1642.45 (1389 to 1920.24)	-36.8 (-47.9 to -19.7)	-38.3 (-53.4 to -17)	-34.4 (-52.1 to -10.3)
	YLLs	2261.37 (1752.12 to 2753.76)	2166.3 (1596.65 to 2704.92)	2325.74 (1705.82 to 3041.65)	1354.95 (1181.71 to 1520.25)	1251.25 (1041.05 to 1468.77)	1466.83 (1212.07 to 1742.13)	-40.1 (-51.3 to -21.3)	-42.2 (-57.6 to -19.1)	-36.9 (-54.8 to -11.3)
	YLDs	219.27 (156.35 to 286.97)	262.27 (187.5 to 339.75)	176.57 (124.41 to 235.14)	212.88 (152.94 to 277.35)	248.19 (176.21 to 326.07)	175.63 (122.95 to 231.45)	-2.9 (-11.3 to 5.1)	-5.4 (-16 to 5.1)	-0.5 (-11 to 11.1)

Province	Measure	Attributed age-standardized rate (per 100,000)						% Change (1990 to 2019)		
		1990			2019					
		Both	Female	Male	Both	Female	Male	Both	Female	Male
Hamadan	Deaths	105.79 (81.73 to 130.26)	109.77 (81.86 to 141.69)	100.08 (68.01 to 129.01)	59.23 (48.43 to 69.14)	62.16 (49.45 to 75.6)	57.88 (45.14 to 70.46)	-44 (-55.9 to -29.5)	-43.4 (-58.3 to -22.9)	-42.2 (-58.8 to -20.5)
	DALYs	2161.48 (1700.45 to 2604.74)	2158.01 (1664.72 to 2740.16)	2143.11 (1549.59 to 2732.16)	1221.14 (1056.67 to 1394.95)	1195.9 (995.34 to 1405.38)	1257.42 (1012.05 to 1506.91)	-43.5 (-55.2 to -30.5)	-44.6 (-57.9 to -26.4)	-41.3 (-57 to -20.7)
	YLLs	1977.88 (1534.78 to 2410.03)	1937.64 (1470.47 to 2522)	1994.35 (1396.76 to 2595.54)	1048.21 (883.45 to 1206.68)	994.17 (809.8 to 1197.23)	1113.56 (876.02 to 1355.04)	-47 (-59.2 to -32.9)	-48.7 (-62.4 to -28.4)	-44.2 (-60.5 to -22.1)
	YLDs	183.59 (131.14 to 237.71)	220.37 (157.72 to 285.42)	148.76 (105.26 to 193.51)	172.93 (122.83 to 226.77)	201.73 (143.54 to 263.47)	143.86 (100.71 to 189.44)	-5.8 (-13 to 2.8)	-8.5 (-17.7 to 2.5)	-3.3 (-12.8 to 8.5)
Hormozgan	Deaths	107.23 (81.45 to 131.3)	97.15 (72.38 to 122.92)	116.36 (80.36 to 152.69)	55.04 (46.13 to 63.13)	56.67 (45.92 to 66.62)	54.95 (43.62 to 66.8)	-48.7 (-59.3 to -32)	-41.7 (-54.7 to -20.6)	-52.8 (-67.1 to -31.4)
	DALYs	2213.09 (1652.19 to 2723.6)	2019.89 (1500.81 to 2516.53)	2371.87 (1654.92 to 3133.24)	1150.1 (1001.12 to 1300.74)	1112.66 (934.44 to 1284.23)	1201.48 (987.18 to 1435.27)	-48 (-58.9 to -29.6)	-44.9 (-57.1 to -24.5)	-49.3 (-63.5 to -25.2)
	YLLs	2024.44 (1458.53 to 2506.26)	1799.4 (1285.25 to 2286.95)	2212.59 (1493.28 to 2954.52)	977.43 (839.28 to 1121.4)	915.61 (755.68 to 1068.4)	1052.9 (847.74 to 1280.69)	-51.7 (-62.6 to -32.2)	-49.1 (-61.4 to -26.9)	-52.4 (-66.6 to -27)
	YLDs	188.65 (134.83 to 244.82)	220.49 (157.22 to 286.73)	159.28 (112.36 to 209.68)	172.67 (123.61 to 226.27)	197.05 (141.11 to 257.72)	148.59 (105.99 to 194.37)	-8.5 (-14.8 to -1.2)	-10.6 (-18.9 to -0.7)	-6.7 (-15.7 to 3.5)
Ilam	Deaths	70.26 (55.53 to 85.19)	62.26 (44.87 to 78.13)	77.15 (59.36 to 99.33)	51.99 (44.45 to 59.62)	49.14 (39.97 to 57.83)	53.99 (45.19 to 63.85)	-26 (-40.2 to -4.8)	-21.1 (-40.3 to 13.8)	-30 (-47.5 to -4.6)
	DALYs	1420.79 (1147.33 to 1707.93)	1311.97 (980.37 to 1619.89)	1506.07 (1171.53 to 1921.07)	1007.88 (892.15 to 1129.81)	973.61 (832.46 to 1123.14)	1033.19 (879.39 to 1206.66)	-29.1 (-42.3 to -9.7)	-25.8 (-43.5 to 3.9)	-31.4 (-48.8 to -7.9)
	YLLs	1275.05 (1005.89 to 1559.44)	1146.83 (812.43 to 1461.97)	1375.92 (1043.84 to 1794.97)	856.39 (750.58 to 966.35)	797.38 (662.56 to 935.59)	905.57 (760.6 to 1078.58)	-32.8 (-46.5 to -11.4)	-30.5 (-48.4 to 1.9)	-34.2 (-52.3 to -8.4)
	YLDs	145.74 (104.24 to 189.98)	165.13 (116.99 to 216.31)	130.16 (93.17 to 171.61)	151.49 (107.78 to 197.91)	176.24 (126.66 to 228.53)	127.61 (89.58 to 167.24)	3.9 (-3.6 to 12.4)	6.7 (-3.4 to 19.1)	-2 (-11.7 to 8.7)

Province	Measure	Attributed age-standardized rate (per 100,000)						% Change (1990 to 2019)		
		1990			2019					
		Both	Female	Male	Both	Female	Male	Both	Female	Male
Isfahan	Deaths	88.83 (68.17 to 110.13)	93.13 (69.44 to 121.99)	81.17 (56.41 to 106.92)	50.29 (41.97 to 59.93)	54.72 (42.84 to 67.69)	46.89 (37.42 to 57.49)	-43.4 (-55.6 to -25.2)	-41.2 (-57.7 to -17.4)	-42.2 (-59.9 to -17.3)
	DALYs	1640.28 (1286 to 2016.46)	1665.67 (1255.89 to 2152.71)	1577.37 (1121.49 to 2067.35)	936.82 (803.97 to 1082.25)	952.95 (792.29 to 1134.8)	928.19 (765.71 to 1107.45)	-42.9 (-55.4 to -25.3)	-42.8 (-58.3 to -21.5)	-41.2 (-58.6 to -16.4)
	YLLs	1460.77 (1103.52 to 1832.08)	1456.67 (1027.64 to 1945.45)	1428.39 (980.96 to 1906.14)	788.89 (668.73 to 922.48)	783.34 (629.07 to 961.46)	801.14 (647.55 to 974.71)	-46 (-59.3 to -26.5)	-46.2 (-62.6 to -22.5)	-43.9 (-62 to -16.8)
	YLDs	179.51 (128.65 to 234.24)	209 (151.14 to 273.11)	148.98 (104.56 to 197.63)	147.93 (105.06 to 190.44)	169.61 (119.93 to 219.46)	127.05 (89.67 to 165)	-17.6 (-23.9 to -10.2)	-18.8 (-26.7 to -9.6)	-14.7 (-22.7 to -6.2)
Kerman	Deaths	98.4 (78.92 to 121.22)	95.87 (73.14 to 121.5)	99.44 (77.6 to 127.06)	53.8 (45.68 to 63.82)	56.05 (45.92 to 67.15)	51.76 (41.94 to 63.36)	-45.3 (-55.8 to -30.8)	-41.5 (-55.9 to -21.9)	-47.9 (-62.2 to -29.6)
	DALYs	1946.26 (1596.06 to 2366.06)	1868.39 (1467.66 to 2359.4)	2000.85 (1581.06 to 2553.19)	1023.77 (888.53 to 1182.02)	1021.69 (863.68 to 1202.12)	1027.08 (856.13 to 1236.16)	-47.4 (-57.2 to -34.9)	-45.3 (-58.8 to -28.5)	-48.7 (-62.5 to -30.4)
	YLLs	1770.66 (1421.31 to 2182.96)	1660.31 (1259.88 to 2179.67)	1856.77 (1436.74 to 2409.01)	877.28 (749.55 to 1025.88)	850.72 (702.97 to 1018.56)	904.61 (737.16 to 1116.6)	-50.5 (-60.9 to -36.9)	-48.8 (-62.7 to -30.8)	-51.3 (-65.2 to -31.7)
	YLDs	175.6 (126.92 to 227.28)	208.08 (148.91 to 269.83)	144.08 (101.86 to 188.13)	146.49 (103.3 to 190.36)	170.97 (122.06 to 223.66)	122.47 (86.74 to 158.14)	-16.6 (-22.9 to -9.6)	-17.8 (-26.5 to -8.7)	-15 (-22.9 to -5.6)
Kermanshah	Deaths	123.68 (96.64 to 149.43)	115.61 (85.56 to 147.64)	129.37 (96.48 to 163.64)	67.43 (56.85 to 78.55)	71.72 (57.79 to 85.39)	64.18 (51.52 to 78.43)	-45.5 (-56.7 to -30.4)	-38 (-54.3 to -14.3)	-50.4 (-63.3 to -32.6)
	DALYs	2532.32 (2029.91 to 3048.44)	2336.34 (1777.21 to 2914.58)	2678.8 (2010.4 to 3389.91)	1339.17 (1156.15 to 1536.98)	1364.25 (1141.65 to 1602)	1322.09 (1083.63 to 1585.06)	-47.1 (-58.3 to -32.6)	-41.6 (-56.1 to -20.3)	-50.6 (-63 to -32.2)
	YLLs	2336.25 (1830.42 to 2847.48)	2104.92 (1540.67 to 2672.2)	2513.35 (1868.24 to 3237.69)	1155.26 (989.57 to 1341.93)	1153.53 (942.55 to 1384.37)	1165.34 (939.65 to 1413.8)	-50.6 (-61.5 to -35.4)	-45.2 (-60.3 to -22.4)	-53.6 (-66.1 to -34.4)
	YLDs	196.07 (139.88 to 254.12)	231.43 (164.66 to 302.27)	165.45 (117.83 to 215.77)	183.9 (131.26 to 240.12)	210.72 (149.54 to 276.44)	156.75 (111.57 to 204.06)	-6.2 (-13.7 to 1.4)	-8.9 (-17.4 to 0.7)	-5.3 (-15.4 to 5.8)

Province	Measure	Attributed age-standardized rate (per 100,000)						% Change (1990 to 2019)		
		1990			2019					
		Both	Female	Male	Both	Female	Male	Both	Female	Male
e-Khorasan- Razavi	Deaths	124.4 (100.78 to 151.2)	117.06 (88.26 to 151.04)	130.99 (101.97 to 169.12)	65.24 (55.28 to 76.4)	64.65 (51.8 to 78.7)	65.95 (53.95 to 80.19)	-47.6 (-58.9 to -33.9)	-44.8 (-58.6 to -24.6)	-49.7 (-63 to -31.6)
	DALYs	2456.5 (2030.75 to 2980.65)	2320.69 (1804.96 to 2979.09)	2572.81 (2006.73 to 3327.92)	1254.37 (1089.2 to 1440.84)	1214.94 (1005.78 to 1442.3)	1295.86 (1083.64 to 1543.73)	-48.9 (-59.6 to -36.9)	-47.6 (-61.3 to -30.5)	-49.6 (-62.9 to -32.1)
	YLLs	2264.45 (1852.36 to 2774.64)	2097.62 (1590.36 to 2731.69)	2410.01 (1850.6 to 3154.2)	1087.95 (930.37 to 1270.76)	1028.75 (834.44 to 1249.29)	1149.69 (946.09 to 1401.3)	-52 (-62.6 to -39.3)	-51 (-65.1 to -32.6)	-52.3 (-65.4 to -34.2)
	YLDs	192.05 (136.19 to 249.87)	223.07 (158.82 to 290.22)	162.8 (114.11 to 214.52)	166.42 (118.16 to 214.57)	186.19 (131.77 to 243.42)	146.17 (101.78 to 188.69)	-13.3 (-19.6 to -7.2)	-16.5 (-24.4 to -8.4)	-10.2 (-18 to -1.1)
Khuzestan	Deaths	108.6 (87.28 to 130.86)	104.78 (80.73 to 132.23)	111.44 (84.81 to 140.15)	72.66 (60.55 to 84.47)	74.74 (59.53 to 89.73)	70.95 (55.86 to 86.98)	-33.1 (-47 to -14.6)	-28.7 (-47 to -4.2)	-36.3 (-53.8 to -12.9)
	DALYs	2140.89 (1766.46 to 2556.02)	2071.02 (1654.83 to 2546.43)	2193.21 (1691.75 to 2760.77)	1394.39 (1205.16 to 1586.54)	1400.5 (1174.07 to 1655.99)	1391.06 (1139.58 to 1661.33)	-34.9 (-47.9 to -19.1)	-32.4 (-49.3 to -11.5)	-36.6 (-53.2 to -14.5)
	YLLs	1936.37 (1567.18 to 2340.12)	1832.23 (1419.59 to 2340.85)	2022.57 (1528.11 to 2573.51)	1190.74 (1016.2 to 1372.25)	1166.89 (943.38 to 1398.79)	1217.64 (968.48 to 1482.23)	-38.5 (-51.9 to -21.7)	-36.3 (-53.6 to -12.6)	-39.8 (-57.1 to -16.1)
	YLDs	204.52 (145.07 to 266.05)	238.79 (168.96 to 309.27)	170.63 (122.23 to 224.87)	203.65 (146.31 to 265.81)	233.61 (167.01 to 305.96)	173.43 (121.72 to 226.4)	-0.4 (-8.8 to 8.4)	-2.2 (-12.6 to 8.7)	1.6 (-9.4 to 13.8)
AhmadandKohgiluyeh Boyer	Deaths	92.96 (71.25 to 115.37)	99.52 (72.78 to 131.93)	83.83 (60.27 to 111.48)	55.19 (44.19 to 67.07)	58.5 (45.5 to 72.8)	53.1 (39.35 to 68.49)	-40.6 (-55.7 to -21.2)	-41.2 (-58.6 to -16.2)	-36.7 (-56.2 to -8.7)
	DALYs	1841.45 (1456.39 to 2251.26)	1917.16 (1453.68 to 2505.96)	1733.93 (1275.19 to 2277.61)	1093.18 (907.79 to 1291.87)	1127.1 (913.22 to 1360.74)	1073.69 (827.38 to 1352.07)	-40.6 (-54.4 to -23.7)	-41.2 (-57.7 to -19.6)	-38.1 (-56.5 to -13)
	YLLs	1665.41 (1281.97 to 2062.35)	1715.22 (1256.87 to 2267.7)	1582.07 (1127.95 to 2100.25)	924.34 (748.88 to 1108.35)	927.93 (722.97 to 1159.47)	931.77 (686.07 to 1207.08)	-44.5 (-58.7 to -26.3)	-45.9 (-62.9 to -22.1)	-41.1 (-60.4 to -13.6)
	YLDs	176.04 (125.31 to 228.78)	201.94 (144.46 to 260.88)	151.86 (107.35 to 199.64)	168.83 (120.4 to 221.13)	199.17 (141.97 to 260.24)	141.92 (101.04 to 186.54)	-4.1 (-10.6 to 4.1)	-1.4 (-10.9 to 9.7)	-6.5 (-15.6 to 4.1)

Province	Measure	Attributed age-standardized rate (per 100,000)						% Change (1990 to 2019)		
		1990			2019					
		Both	Female	Male	Both	Female	Male	Both	Female	Male
Kurdistan	Deaths	107.17 (86 to 127.28)	98.11 (74.79 to 123.99)	115.19 (87.9 to 144.75)	52.66 (44.33 to 60.41)	53.63 (43.49 to 63.57)	52.49 (42.17 to 64.23)	-50.9 (-60.9 to -38)	-45.3 (-58.9 to -27)	-54.4 (-67 to -37.8)
	DALYs	2175.96 (1794.28 to 2574.51)	2035.11 (1598.9 to 2545.7)	2290.06 (1788.08 to 2882.88)	1062.76 (928.24 to 1206.52)	1037.59 (873.79 to 1207.8)	1095.5 (903.3 to 1313.87)	-51.2 (-60.7 to -39.2)	-49 (-61.6 to -34)	-52.2 (-65.1 to -35.4)
	YLLs	2001.99 (1633.27 to 2406.5)	1832.67 (1408.39 to 2348.53)	2140.77 (1633.09 to 2725.24)	903.04 (778.1 to 1038.56)	855.76 (701.72 to 1009.11)	957.44 (774.58 to 1171.25)	-54.9 (-64.6 to -42.8)	-53.3 (-65.8 to -37)	-55.3 (-68.4 to -37.7)
	YLDs	173.97 (123.44 to 226.27)	202.44 (142.37 to 262.44)	149.29 (105.71 to 195.15)	159.72 (113.16 to 205.54)	181.83 (128.21 to 235.94)	138.06 (97.44 to 179.52)	-8.2 (-15.2 to -1.2)	-10.2 (-19.3 to -0.1)	-7.5 (-16.6 to 3.4)
Lorestan	Deaths	113.35 (89.86 to 137.97)	102.64 (75.65 to 130.16)	122.51 (89.42 to 158.1)	67.77 (54.07 to 80.13)	55.81 (41.91 to 67.28)	80.1 (60.66 to 97.87)	-40.2 (-53.4 to -24.1)	-45.6 (-61.1 to -24)	-34.6 (-53.4 to -10.2)
	DALYs	2266.47 (1805.62 to 2759.75)	2052.42 (1566.55 to 2582.52)	2439.92 (1828.55 to 3163.75)	1324.48 (1098.71 to 1542.85)	1097.72 (869.95 to 1305.83)	1563.2 (1225.55 to 1892.45)	-41.6 (-53.7 to -25.8)	-46.5 (-61.6 to -28.2)	-35.9 (-53.1 to -12.1)
	YLLs	2081.09 (1629.5 to 2553.76)	1837.94 (1342.01 to 2359.94)	2280.59 (1656.06 to 2994.19)	1154.27 (940.73 to 1361.67)	908.1 (682.21 to 1105.13)	1412.87 (1083.3 to 1735.83)	-44.5 (-57.1 to -27.5)	-50.6 (-66.2 to -30.7)	-38 (-55.7 to -11.8)
	YLDs	185.38 (133.19 to 239.17)	214.47 (155.3 to 278.85)	159.34 (113.38 to 207.34)	170.22 (121.73 to 220.94)	189.62 (135.53 to 246.61)	150.32 (108.03 to 197.89)	-8.2 (-15.2 to -0.9)	-11.6 (-20 to -2.4)	-5.7 (-15.7 to 4.5)
Markazi	Deaths	109.85 (89.19 to 133.9)	109.16 (82.18 to 138.86)	109.05 (82.37 to 138.01)	50.76 (42.39 to 60.11)	52.45 (41.98 to 64.92)	49.49 (39.47 to 60.97)	-53.8 (-63.8 to -40.5)	-52 (-64.2 to -32.9)	-54.6 (-66.6 to -37)
	DALYs	2152.04 (1795.83 to 2581.56)	2081.24 (1621.16 to 2605.97)	2204.39 (1685.47 to 2799.25)	1016.95 (877.76 to 1177.3)	1002.92 (844.68 to 1203.37)	1032.34 (845.42 to 1246.22)	-52.7 (-62.9 to -40.9)	-51.8 (-64 to -34.8)	-53.2 (-65.3 to -35.7)
	YLLs	1958.41 (1603.45 to 2393.79)	1854.32 (1398.19 to 2366.68)	2043.8 (1543.65 to 2646.98)	852.52 (720.87 to 1000.78)	814.18 (661.53 to 1006.56)	892.76 (716.97 to 1104.45)	-56.5 (-66.6 to -44.1)	-56.1 (-68 to -38)	-56.3 (-68.8 to -37.8)
	YLDs	193.63 (137.03 to 249.89)	226.91 (161.72 to 292.85)	160.59 (114.94 to 210.82)	164.43 (116.55 to 212.81)	188.73 (133.78 to 244.1)	139.58 (98.36 to 182.12)	-15.1 (-21.7 to -8)	-16.8 (-25.3 to -8.1)	-13.1 (-21.7 to -2.1)

Province	Measure	Attributed age-standardized rate (per 100,000)						% Change (1990 to 2019)		
		1990			2019					
		Both	Female	Male	Both	Female	Male	Both	Female	Male
Mazandaran	Deaths	95.49 (76.31 to 116.4)	100.4 (77.51 to 125.55)	88.31 (64.56 to 113.91)	54.05 (45.32 to 63.49)	56.06 (44.96 to 68.33)	51.98 (41.48 to 63.22)	-43.4 (-54.9 to -27.2)	-44.2 (-58.2 to -23)	-41.1 (-57.7 to -16.6)
	DALYs	1863.87 (1528.65 to 2230.4)	1954.19 (1551.47 to 2408.12)	1751.91 (1338.79 to 2245.34)	1091.74 (949.88 to 1244.59)	1104.18 (920.36 to 1304.71)	1077.6 (886.89 to 1296.04)	-41.4 (-52.6 to -25.9)	-43.5 (-56.9 to -23.8)	-38.5 (-54.9 to -15.3)
	YLLs	1660.4 (1332.72 to 2025.33)	1715.25 (1324.54 to 2183.26)	1584.37 (1170.65 to 2073.1)	911.88 (779.79 to 1059.1)	897.64 (728.92 to 1090.65)	925.1 (749.55 to 1126.32)	-45.1 (-56.8 to -28.3)	-47.7 (-61.8 to -26.7)	-41.6 (-58.8 to -16.2)
	YLDs	203.47 (145.32 to 265.34)	238.94 (169.04 to 311.53)	167.53 (119.26 to 220.28)	179.86 (128.33 to 232.03)	206.54 (146.4 to 268.22)	152.5 (108.03 to 197.4)	-11.6 (-18.1 to -4.4)	-13.6 (-21.8 to -3.7)	-9 (-17.4 to 0.9)
North Khorasan	Deaths	117.47 (91.31 to 145.1)	116.11 (84.23 to 151.41)	117.45 (88.96 to 156.7)	60.79 (51.8 to 71.5)	65.8 (53.54 to 79.43)	57.08 (47.22 to 69.01)	-48.3 (-58.8 to -33)	-43.3 (-57.7 to -19.9)	-51.4 (-65.1 to -33.4)
	DALYs	2405.78 (1953.74 to 2890.77)	2387.43 (1825.04 to 3042.28)	2402.99 (1851.1 to 3144.62)	1245.06 (1088.05 to 1435.36)	1313.22 (1106.47 to 1554.29)	1188.81 (1003.7 to 1427.6)	-48.2 (-58 to -35.9)	-45 (-57.7 to -26.4)	-50.5 (-64 to -32.7)
	YLLs	2212.79 (1763.03 to 2717.99)	2156.43 (1595.46 to 2818.78)	2245.63 (1689.48 to 2993.28)	1067.14 (920.79 to 1248.44)	1101.29 (911.95 to 1328.51)	1045.04 (866.82 to 1272.08)	-51.8 (-62.1 to -38.9)	-48.9 (-62 to -29.6)	-53.5 (-67 to -34.7)
	YLDs	192.99 (139.02 to 249.78)	231 (167.22 to 296.76)	157.36 (110.78 to 208.42)	177.93 (127.41 to 231.9)	211.93 (150.02 to 278.09)	143.77 (102.23 to 188.97)	-7.8 (-14.6 to -0.6)	-8.3 (-17.5 to 3)	-8.6 (-17.4 to 2)
Qazvin	Deaths	86.56 (66.41 to 106.89)	83.39 (59.85 to 109.26)	90.05 (66 to 120.51)	62.67 (51.47 to 73.64)	53.46 (41.49 to 64.55)	72.59 (58.16 to 85.86)	-27.6 (-45.2 to -4.3)	-35.9 (-53.5 to -7.2)	-19.4 (-43.4 to 10.9)
	DALYs	1737.6 (1371.42 to 2135.43)	1702.96 (1268.03 to 2162.41)	1768.42 (1315.54 to 2372.83)	1184.61 (1016.37 to 1348.67)	1014.44 (837.45 to 1190.66)	1364.3 (1146.56 to 1594.24)	-31.8 (-48.1 to -12.6)	-40.4 (-55.3 to -18.4)	-22.9 (-46 to 6.2)
	YLLs	1576.09 (1217.72 to 1965.84)	1514.83 (1089.91 to 1975.58)	1633.02 (1187.46 to 2235.02)	1023.32 (860.4 to 1186.32)	833.54 (669.34 to 1011.77)	1223.56 (1001.58 to 1443.87)	-35.1 (-52.2 to -14.5)	-45 (-60.7 to -21.2)	-25.1 (-49 to 6.6)
	YLDs	161.5 (115.12 to 209.03)	188.13 (132.81 to 246.82)	135.4 (96.93 to 176.28)	161.29 (113.87 to 208.77)	180.9 (126.71 to 236.13)	140.74 (98.84 to 183.28)	-0.1 (-7.4 to 7.7)	-3.8 (-13.4 to 6.7)	3.9 (-6.1 to 14.8)

Province	Measure	Attributed age-standardized rate (per 100,000)						% Change (1990 to 2019)		
		1990			2019					
		Both	Female	Male	Both	Female	Male	Both	Female	Male
Qom	Deaths	117.19 (85.92 to 148.14)	120.63 (84.51 to 157.46)	112.45 (78.47 to 148.75)	50.24 (42.1 to 58.47)	57.83 (46.63 to 68.42)	45.71 (36.72 to 55.77)	-57.1 (-66.9 to -42.3)	-52.1 (-64.4 to -31.3)	-59.3 (-70.9 to -41)
	DALYs	2215.27 (1677.78 to 2783.32)	2230.15 (1587.91 to 2889.21)	2182.8 (1577.78 to 2889.17)	974.21 (842.85 to 1106.57)	1039.92 (866.99 to 1202.83)	931.91 (766.94 to 1114.85)	-56 (-66 to -42.6)	-53.4 (-65.3 to -33.7)	-57.3 (-69.1 to -38.5)
	YLLs	2030.11 (1501.33 to 2600.42)	2014.37 (1397.93 to 2667.27)	2027.01 (1405.56 to 2735.85)	823.97 (700.12 to 945.69)	863.59 (709.65 to 1016.73)	805.53 (649.36 to 980.86)	-59.4 (-69.4 to -45.5)	-57.1 (-69.4 to -36.2)	-60.3 (-71.8 to -40.8)
	YLDs	185.16 (131.65 to 240.55)	215.78 (151.87 to 279.34)	155.79 (111.24 to 205.27)	150.25 (105.76 to 196.03)	176.32 (123.52 to 230.98)	126.38 (88.72 to 165.38)	-18.9 (-24.6 to -12.2)	-18.3 (-26.4 to -8.6)	-18.9 (-26.6 to -9.6)
Semnan	Deaths	101.3 (79.26 to 126.6)	94.35 (67.74 to 123.51)	109.22 (82.28 to 141.63)	60.81 (50.4 to 72.07)	52.61 (41.01 to 64.49)	69.63 (57.62 to 82.64)	-40 (-52.1 to -22)	-44.2 (-59.6 to -20.4)	-36.2 (-52.4 to -13.6)
	DALYs	2013.03 (1608.48 to 2480.65)	1876.2 (1426.02 to 2437.11)	2153.13 (1626.63 to 2782.95)	1126.34 (973.24 to 1294.73)	982.35 (805.19 to 1160.33)	1276.57 (1087.62 to 1481.68)	-44 (-54.6 to -29.5)	-47.6 (-60.6 to -28.9)	-40.7 (-54.9 to -20.7)
	YLLs	1813.86 (1406.66 to 2268.82)	1649.9 (1172.46 to 2174.76)	1982.31 (1466.52 to 2580.41)	948.8 (806.23 to 1107.53)	781.58 (621.76 to 951.86)	1123.34 (942.3 to 1325.95)	-47.7 (-58.8 to -31.6)	-52.6 (-66.3 to -32.4)	-43.3 (-58.1 to -21.6)
	YLDs	199.17 (140 to 258.38)	226.3 (159.16 to 293.34)	170.82 (119.67 to 225.13)	177.54 (127.17 to 229.83)	200.78 (143.76 to 262.06)	153.23 (107.88 to 199.17)	-10.9 (-17.4 to -2.8)	-11.3 (-20.4 to -1.1)	-10.3 (-18.8 to -0.5)
Sistan and Baluchistan	Deaths	89.46 (67.18 to 111.25)	79.61 (55.82 to 106.04)	98.19 (70.18 to 126.85)	52.21 (42.61 to 63.73)	48.64 (37.73 to 60.49)	55.88 (43.35 to 71.45)	-41.6 (-56.2 to -23.3)	-38.9 (-56.9 to -8.8)	-43.1 (-60.3 to -18.2)
	DALYs	1855.67 (1389.6 to 2317.28)	1694.1 (1230.76 to 2222.83)	1982.67 (1356.38 to 2605.61)	1201.84 (1014.6 to 1430.74)	1117.77 (905.71 to 1359.81)	1288.04 (1031 to 1611.28)	-35.2 (-51.4 to -13)	-34 (-52.5 to -5.3)	-35 (-54.5 to -2.9)
	YLLs	1695.21 (1203.55 to 2155.49)	1506.78 (1053.15 to 2020.12)	1844.82 (1229.09 to 2457.63)	1052.59 (859.4 to 1271.26)	948.44 (737.85 to 1182.39)	1158.6 (899.94 to 1472.44)	-37.9 (-54.2 to -13.3)	-37.1 (-56.8 to -4.6)	-37.2 (-57.2 to -2.6)
	YLDs	160.46 (113.6 to 207.91)	187.32 (132.66 to 241.21)	137.85 (97.32 to 179.91)	149.25 (106.86 to 192.62)	169.33 (122.25 to 218.32)	129.44 (92.14 to 169.36)	-7 (-13.5 to 0.6)	-9.6 (-17.9 to -0.1)	-6.1 (-15.3 to 3.7)

Province	Measure	Attributed age-standardized rate (per 100,000)						% Change (1990 to 2019)		
		1990			2019					
		Both	Female	Male	Both	Female	Male	Both	Female	Male
South Khorasan	Deaths	85.07 (68 to 104.95)	82.36 (60.48 to 106.22)	87.61 (68.36 to 111.14)	48.6 (40.57 to 57.2)	46.39 (37.16 to 56.51)	51.61 (42.91 to 61.95)	-42.9 (-53.7 to -27.7)	-43.7 (-56.9 to -22.2)	-41.1 (-55.6 to -22.1)
	DALYs	1675.44 (1373.53 to 2038.84)	1641.07 (1270.41 to 2067.74)	1705.93 (1326.9 to 2184.52)	940.77 (810.94 to 1082.13)	911.57 (762.24 to 1065.25)	976.32 (827.5 to 1169.95)	-43.8 (-54.2 to -31.3)	-44.5 (-56.9 to -27.4)	-42.8 (-56.3 to -24.7)
	YLLs	1508.34 (1214.09 to 1876.73)	1443.55 (1090.12 to 1870.5)	1567.01 (1202.18 to 2051.47)	786.62 (672.25 to 916.04)	737.84 (602.97 to 893.2)	842.68 (700.15 to 1032.56)	-47.8 (-58.6 to -34.6)	-48.9 (-62.2 to -30)	-46.2 (-59.8 to -26.9)
	YLDs	167.11 (118.86 to 216.4)	197.52 (140.69 to 257.79)	138.91 (99.4 to 181.91)	154.16 (109.92 to 200.13)	173.72 (123.83 to 227.29)	133.64 (94.27 to 173.82)	-7.7 (-13.9 to -0.6)	-12.1 (-20.9 to -2.3)	-3.8 (-12.5 to 5.7)
Tehran	Deaths	53.88 (41.82 to 69.36)	55.34 (39.66 to 73.53)	51.26 (37.51 to 68.98)	30.49 (24.87 to 36.79)	34.19 (26.65 to 42.95)	27.46 (21.45 to 35.01)	-43.4 (-57.9 to -24.6)	-38.2 (-58.2 to -12.8)	-46.4 (-62.6 to -23.2)
	DALYs	1071.61 (860.57 to 1321.94)	1053.74 (806.11 to 1343.78)	1070.35 (812.24 to 1395.78)	622.78 (534.14 to 723.94)	650.98 (532.08 to 792.11)	600.17 (489.81 to 730.28)	-41.9 (-53.9 to -26.8)	-38.2 (-54.9 to -17.7)	-43.9 (-59.5 to -22.6)
	YLLs	908 (707.61 to 1149.88)	869.27 (634.21 to 1149.38)	927.79 (680.81 to 1256.55)	481.59 (399.73 to 575.51)	492.71 (387.17 to 617.35)	475.65 (374.43 to 602.22)	-47 (-60.3 to -29.2)	-43.3 (-61.6 to -18.9)	-48.7 (-65.3 to -24.5)
	YLDs	163.61 (117.34 to 212.02)	184.47 (131.06 to 237.88)	142.57 (101.9 to 187.66)	141.19 (100.45 to 183.83)	158.27 (113.2 to 205.26)	124.52 (87.45 to 161.63)	-13.7 (-19.2 to -7.3)	-14.2 (-22 to -5.1)	-12.7 (-21.4 to -3.2)
West Azarbayejan	Deaths	109.8 (89.02 to 133.13)	109.99 (83.32 to 138.84)	108.34 (83.89 to 134.64)	70.38 (58.59 to 82.45)	71.9 (58.75 to 87.29)	68.71 (55.55 to 82.7)	-35.9 (-48.5 to -19.9)	-34.6 (-49.8 to -10.8)	-36.6 (-52.6 to -15.9)
	DALYs	2076.23 (1724.35 to 2477.23)	2076.72 (1631 to 2593.29)	2062.16 (1622.4 to 2576.19)	1262.53 (1090.44 to 1439.47)	1254.12 (1050.18 to 1470.35)	1267.83 (1047.68 to 1505.8)	-39.2 (-50.6 to -25.1)	-39.6 (-53.6 to -21.1)	-38.5 (-53.9 to -18.6)
	YLLs	1888.1 (1546.71 to 2288.55)	1851.33 (1413.71 to 2351.19)	1908.77 (1473.1 to 2416.32)	1090.38 (923.76 to 1257.27)	1054.6 (872.35 to 1265.28)	1124.61 (906.33 to 1363.31)	-42.2 (-54.5 to -27.4)	-43 (-57.7 to -22.6)	-41.1 (-57.2 to -19.6)
	YLDs	188.13 (133.62 to 243.63)	225.4 (160.99 to 290.5)	153.39 (107.15 to 202.81)	172.15 (122.18 to 225.28)	199.52 (141.47 to 258.86)	143.22 (101.45 to 187.98)	-8.5 (-15.3 to -0.8)	-11.5 (-20.6 to -1.3)	-6.6 (-16.3 to 4.2)

Province	Measure	Attributed age-standardized rate (per 100,000)						% Change (1990 to 2019)		
		1990			2019					
		Both	Female	Male	Both	Female	Male	Both	Female	Male
Yazd	Deaths	106.35 (81.68 to 132.48)	105.57 (74.46 to 136.01)	105.36 (78.78 to 137.48)	63.35 (51.73 to 75.78)	63.02 (49.74 to 78.37)	63.58 (50.26 to 79.73)	-40.4 (-53.1 to -22.2)	-40.3 (-56.4 to -16.5)	-39.7 (-56.4 to -16)
	DALYs	1971.88 (1582.58 to 2400.59)	1956.82 (1456.27 to 2493.87)	1973.55 (1497.77 to 2537.84)	1116.6 (944.06 to 1296.5)	1093 (889.58 to 1320.09)	1134.77 (920.95 to 1386.99)	-43.4 (-55 to -28.1)	-44.1 (-58.4 to -24.4)	-42.5 (-57.6 to -19.2)
	YLLs	1793.21 (1400.8 to 2218.15)	1750.96 (1251.54 to 2263.26)	1823.49 (1350.3 to 2398.34)	953.35 (791.43 to 1129.07)	905.42 (726.53 to 1128.89)	995.71 (781.87 to 1240.63)	-46.8 (-59.8 to -30.5)	-48.3 (-63.3 to -26.8)	-45.4 (-61.2 to -20.9)
	YLDs	178.67 (128.2 to 235.53)	205.86 (146.62 to 270.42)	150.07 (105.52 to 200.99)	163.25 (116.97 to 211.47)	187.58 (135.46 to 243.7)	139.06 (97.22 to 183.68)	-8.6 (-15.4 to -1.8)	-8.9 (-17.6 to 1)	-7.3 (-16.3 to 2.6)
Zanjan	Deaths	88.86 (70.52 to 110.51)	79.4 (58.73 to 103.77)	99.02 (72.81 to 125.3)	57.46 (47.74 to 65.73)	49.2 (39.29 to 58.09)	66.66 (54.73 to 78.07)	-35.3 (-48.2 to -18.9)	-38 (-53.4 to -14.9)	-32.7 (-49.6 to -11.3)
	DALYs	1810.66 (1477.84 to 2217.66)	1626.18 (1277.89 to 2054.3)	1983.1 (1549.05 to 2502.77)	1100.01 (964.41 to 1236.89)	954.59 (805.8 to 1101.93)	1257.02 (1076.16 to 1451.54)	-39.2 (-51.4 to -25.5)	-41.3 (-54.4 to -23.5)	-36.6 (-52.1 to -16.3)
	YLLs	1622.84 (1295.4 to 2026.89)	1413.29 (1065.43 to 1849.26)	1820.22 (1393.88 to 2344.79)	932.41 (800.1 to 1060.1)	763.87 (624.41 to 895.37)	1113.89 (937.49 to 1309.92)	-42.5 (-55.4 to -27.8)	-46 (-60.3 to -26.2)	-38.8 (-54.9 to -17.2)
	YLDs	187.82 (134.26 to 244.85)	212.89 (150.64 to 278.95)	162.88 (116.51 to 213.77)	167.6 (118.63 to 216.27)	190.72 (135.17 to 245.21)	143.13 (99.71 to 187.13)	-10.8 (-17.2 to -3.7)	-10.4 (-19.2 to -1.3)	-12.1 (-21 to -1.2)

Data in parentheses are 95% Uncertainty Intervals (95% UIs)
